# The anti-immune dengue subgenomic flaviviral RNA is present in vesicles in mosquito saliva and is associated with increased infectivity

**DOI:** 10.1371/journal.ppat.1011224

**Published:** 2023-03-30

**Authors:** Shih-Chia Yeh, Tania Strilets, Wei-Lian Tan, David Castillo, Hacène Medkour, Félix Rey-Cadilhac, Idalba M. Serrato-Pomar, Florian Rachenne, Avisha Chowdhury, Vanessa Chuo, Sasha R. Azar, Moirangthem Kiran Singh, Rodolphe Hamel, Dorothée Missé, R. Manjunatha Kini, Linda J. Kenney, Nikos Vasilakis, Marc A. Marti-Renom, Guy Nir, Julien Pompon, Mariano A. Garcia-Blanco

**Affiliations:** 1 Programme in Emerging Infectious Diseases, Duke-NUS Medical School, Singapore; 2 Department of Biochemistry and Molecular Biology, University of Texas Medical Branch, Galveston, Texas, United States of America; 3 CNAG-CRG, Centre for Genomic Regulation (CRG), Barcelona Institute of Science and Technology (BIST), Barcelona, Spain; 4 MIVEGEC, Univ. Montpellier, IRD, CNRS, Montpellier, France; 5 Department of Biological Sciences, National University of Singapore, Singapore; 6 Department of Pathology, University of Texas Medical Branch, Galveston, Texas, United States of America; 7 Department of Pharmacology, Yong Loo Lin School of Medicine, National University of Singapore, Singapore; 8 Center for Biodefense and Emerging Infectious Diseases, University of University of Texas Medical Branch, Galveston, Texas, United States of America; 9 Center for Tropical Diseases, University of University of Texas Medical Branch, Galveston, Texas, United States of America; 10 Institute for Human Infection and Immunity, University of University of Texas Medical Branch, Galveston, Texas, United States of America; 11 Center for Vector-Borne and Zoonotic Diseases, University of University of Texas Medical Branch, Galveston, Texas, United States of America; 12 Department of Preventive Medicine and Population Health, University of University of Texas Medical Branch, Galveston, Texas, United States of America; 13 World Reference Center for Emerging Viruses and Arboviruses, University of University of Texas Medical Branch, Galveston, Texas, United States of America; 14 Centre for Genomic Regulation (CRG), Barcelona Institute of Science and Technology (BIST), Barcelona, Spain; 15 Universitat Pompeu Fabra (UPF), Barcelona, Spain; 16 ICREA, Barcelona, Spain; 17 Department of Internal Medicine, University of Texas Medical Branch, Galveston, Texas, United States of America; 18 Department of Microbiology, Immunology and Cancer Biology, University of Virginia, Charlottesville, Virginia, United States of America; University of Pittsburgh, UNITED STATES

## Abstract

Mosquito transmission of dengue viruses to humans starts with infection of skin resident cells at the biting site. There is great interest in identifying transmission-enhancing factors in mosquito saliva in order to counteract them. Here we report the discovery of high levels of the anti-immune subgenomic flaviviral RNA (sfRNA) in dengue virus 2-infected mosquito saliva. We established that sfRNA is present in saliva using three different methods: northern blot, RT-qPCR and RNA sequencing. We next show that salivary sfRNA is protected in detergent-sensitive compartments, likely extracellular vesicles. In support of this hypothesis, we visualized viral RNAs in vesicles in mosquito saliva and noted a marked enrichment of signal from 3’UTR sequences, which is consistent with the presence of sfRNA. Furthermore, we show that incubation with mosquito saliva containing higher sfRNA levels results in higher virus infectivity in a human hepatoma cell line and human primary dermal fibroblasts. Transfection of 3’UTR RNA prior to DENV2 infection inhibited type I and III interferon induction and signaling, and enhanced viral replication. Therefore, we posit that sfRNA present in salivary extracellular vesicles is delivered to cells at the biting site to inhibit innate immunity and enhance dengue virus transmission.

## Author summary

Mosquitoes transmit many viruses, such as those that cause dengue, introducing them in our bodies with the saliva that is deposited in our skins when they bite us. Scientists who study biting behavior and properties of mosquito saliva have concluded that there may be components in saliva that promote virus infection. In this study we show that when dengue virus infects mosquitoes one can find in their saliva not only the expected dengue viruses but also a dengue virus product that can reduce our anti-virus defense systems. It happens that this product is what is known as a non-coding RNA, a class of molecules that have recently been shown to mediate important biological regulation. We propose that by introducing this RNA at the biting site dengue infected saliva prepares the terrain for an efficient infection and gives the virus an advantage in the first battle between it and our immune defenses.

## Introduction

Half of the world’s human population is at risk of infection with dengue viruses (DENV) transmitted primarily by *Aedes aegypti* mosquitoes [1]. Since DENV have evolved to harness mosquito biology to enhance transmission to humans, understanding the transmission cycle of DENV is important for controlling the spread of this disease. It has become clear that mosquito saliva enhances infection in human cells [2], mouse models [3], and non-human primates [4]. The mechanistic underpinnings for this enhancement are beginning to be understood [5,6], and here we propose that the subgenomic flaviviral RNA (sfRNA) is a novel transmission enhancer.

sfRNA is a noncoding RNA (ncRNA) produced from partial degradation of the flaviviral RNA genome (gRNA) by host 5’-3’ exoribonucleases that stall at secondary structures in the 3’ untranslated region (UTR) [7]. Data from many groups including ours demonstrate that sfRNA downregulates immune responses in both human and insect cells [8–10] and therefore we refer to this noncoding RNA as an anti-immune mediator. For instance, DENV serotype 2 (DENV2) sfRNA inhibits the innate immune response by limiting interferon (IFN) expression [11] and translation of IFN stimulated gene products [8], promoting DENV2 propagation in human cells. Moreover, Zika virus (ZIKV) sfRNA can modulate mRNA decay and splicing and likely limit the effective response of cells to viral infection [12]. In DENV2-infected mosquitoes, sfRNA downregulates expression of Toll pathway-associated genes *Rel1a* and *CecG* [13]. In mosquitoes infected with ZIKV, sfRNA suppresses apoptosis in mosquito tissues and this enhances virus dissemination and accumulation in saliva [14]. Strong evidence of the epidemiological function of sfRNA comes from studies that show that DENV2 strains with higher sfRNA levels, in both human and mosquito, have higher epidemiological fitness [11,13,15]. Higher sfRNA quantity may result from either increased production or increased stability. Importantly, we had previously noted that DENV2 sfRNA is expressed at very high levels in salivary glands [13]. Here, we demonstrate that sfRNA is secreted into the saliva of DENV2-infected mosquitoes and is found in detergent-sensitive compartments, which we posit are extracellular vesicles. Importantly, our functional data reveal that levels of salivary sfRNA positively correlate with efficiency of DENV2 replication and that transfection of *in vitro*-transcribed sfRNA inhibits interferon induction and enhances viral replication. Based on these data we propose that sfRNA present in salivary extracellular vesicles is delivered to cells at the bite site to inhibit innate immunity, enhance skin infection and increase dengue virus transmission.

## Results

### DENV2-infected mosquito saliva contains high levels of subgenomic flaviviral RNA

**Fig 1:**
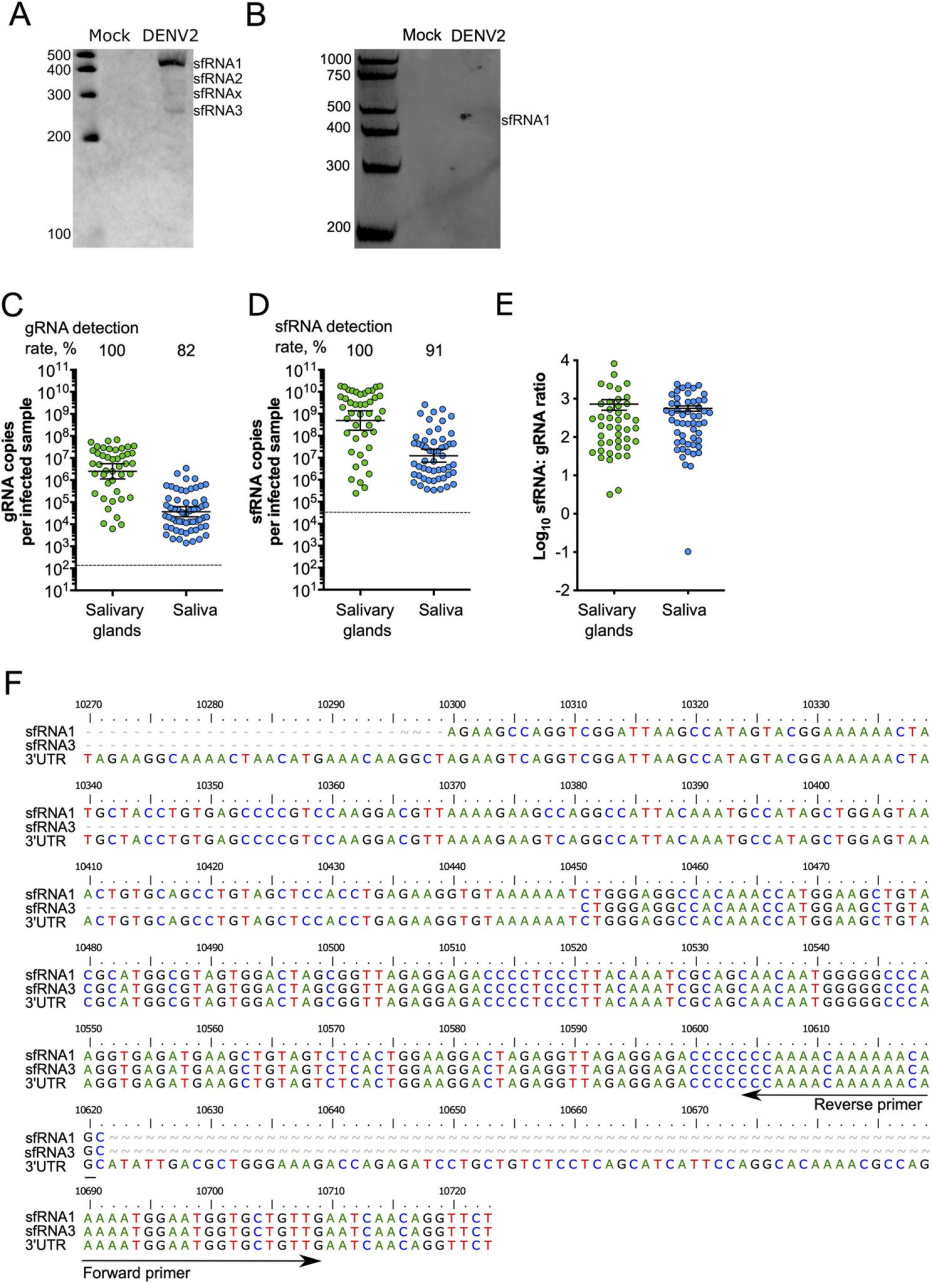
Detection of sfRNA in mosquito saliva. (**A**) sfRNA species detected by Northern blotting in infected mosquitoes. **(B)** sfRNA1 detected by northern blotting in infected pooled mosquito saliva. (**C**) gRNA; (**D**) sfRNA; and (**E**) sfRNA:gRNA ratio in salivary glands and saliva determined by qRT-PCR. Points represent a pair of salivary glands, or one saliva collected from one mosquito. (**C**-**D**) Bars represent geometric means ± 95% C.I. and dashed lines indicate limit of detections which were 133 copies in gRNA (**C**) and 31,216 copies in 3’ UTR/ sfRNA1 (**D**). (**E)** Bars represent arithmetic means ± s.e.m. N salivary glands, 43; N saliva, 71; from two mosquito batches. (**F**) Sequences of sfRNA species found in saliva.

There are several species of DENV2 sfRNA produced by differential exonuclease stalling in mosquito cells and mosquitoes [16]. In RNA extracted from whole *Ae*. *aegypti* mosquitoes orally infected with DENV2 NGC strain, we detected three of the expected species, sfRNA1-3, and one additional species, sfRNAx (**Fig 1A**). To test whether sfRNA is secreted in mosquito saliva, we analyzed RNA extracted from pooled saliva from *Ae*. *aegypti* inoculated with DENV2 NGC using northern blots and detected sfRNA1 **(Fig 1B)**. Since sfRNA1 was the major species detected in whole mosquitoes and saliva, we quantified sfRNA1 copies using RT-qPCR as previously described [13]. To account for variation in RT and qPCR efficiency when measuring sfRNA and gRNA, we calculated the absolute number of sfRNA and gRNA copies by using standard curves generated from *in vitro*-transcribed sfRNA and gRNA (**S1 and S2 Figs**). Moreover, we separately calculated the detection rates and quantities in positive samples to differentiate between two phenomena. We interpret gRNA rates as a measure of infection initiation, and sfRNA rates as an indication for successful initiation of sfRNA production. We interpret gRNA quantities as an indicator of the virus ability to replicate and sfRNA quantities as an information about sfRNA production process and stability. At 10 days post-oral infection (d.p.i.), gRNA was present in salivary glands and in saliva (geometric mean of 3.6 x 10^4^ copies per infected saliva expectorated by one mosquito, which we refer to as saliva sample) (**Fig 1C**). We detected sfRNA1 in 100% of infected salivary glands and, importantly, in 91% of infected saliva samples (geometric mean of 1.1 x 10^7^ sfRNA copies per infected saliva sample; 95% CI; lower = 5.6 x 10^6^; upper = 2.2 x 10^7^) (**Fig 1D**). To normalize sfRNA to its precursor and infection level (both estimated by the copies of gRNA), we calculated sfRNA:gRNA ratios. The ratio in salivary glands was 720 (± 221.5) and not significantly different from the ratio in saliva, which was 559.2 (± 89.07) (t-test; p = 0.47) (**Fig 1E**). We also detected sfRNA in saliva from mosquitoes orally infected with three other DENV2 strains: PR1940, PR6452, and PR9963 (**S3 Fig**), which supports the generality of salivary sfRNA. Of note, sfRNA;gRNA ratios vary between virus strains (from 26 to 559) and this may be related to genetic variations between strains [10,11,13,17] or to variable mosquito biology [18].

To validate the presence of sfRNA in infected saliva with an independent approach, we used the well-established method of RNA circularization and sequencing to determine the sequence of sfRNA species [16]. We detected sfRNA1 and sfRNA3 sequences in DENV2 NGC infected mosquitoes (**S4 Fig**) and in saliva (**Figs 1F and S5**). Together, these results led us to conclude that sfRNA, which is a potent immune inhibitor, is present in the saliva of DENV2-infected mosquitoes.

### sfRNA is present in detergent-sensitive compartments in mosquito saliva

**Fig 2:**
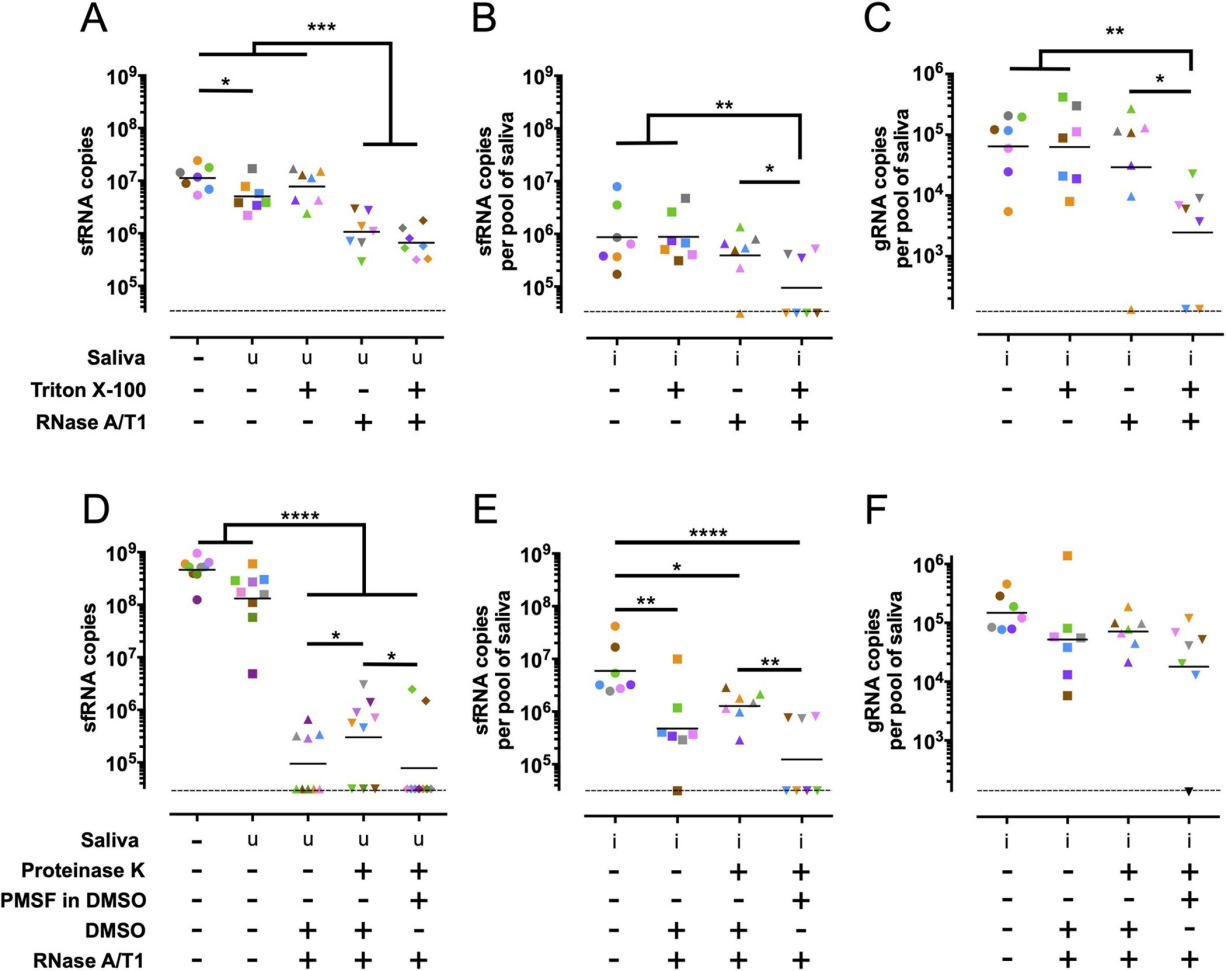
Salivary sfRNA is sensitive to nuclease degradation only after detergent treatment. (**A**-**C**) Impact of Triton X-100 treatment on nuclease resistance for *in vitro* transcribed sfRNA1 in uninfected saliva (**A**) and for sfRNA1 (**B**) and gRNA (**C**) from infected saliva. (**D**-**F**) Impact of DMSO and Proteinase K treatment on nuclease resistance for *in vitro* transcribed sfRNA1 in uninfected saliva (**D**) and for sfRNA1 (**E**) and gRNA (**F**) from infected saliva. Compare the third and fourth group in **E** to note that unless inhibited by PMSF PK decreased RNase activity. Symbol colors indicate biological repeats. Saliva samples were collected from three mosquito batches. Dashed lines indicate limits of detection which were 133 copies in gRNA and 31,216 copies in 3’ UTR/ sfRNA1. u, uninfected. i, infected. *, p-value < 0.05; **, p-value < 0.01; ***, p-value < 0.001, ****, p-value <0.0001 as determined by Fisher’s LSD test.

Extracellular ncRNAs can be packaged in extracellular vesicles (EVs), including microvesicles and exosomes [19], or in ribonucleoprotein complexes with RNA binding proteins [20]. Therefore, to determine how sfRNA is secreted and protected in saliva, we subjected infected saliva to different biochemical treatments and tested their effect on sfRNA levels. We used *in vitro*-synthesized sfRNA1, which we added to uninfected mosquito saliva, as a control for these experiments. sfRNA1 incubated in uninfected saliva was relatively stable, but as expected this RNA could be digested by adding RNases A/T1 (**Fig 2A**). In contrast, the sfRNA1 found in infected saliva was resistant to RNase treatment (**Fig 2B**). If the infected saliva was pre-treated with Triton X-100, however, the great majority of the sfRNA in saliva was digested by RNase (**Fig 2B**). Of note, each color represents one independent repeat in the RNase treatment. One out of seven samples (pink) did not show decreased sfRNA levels and this might be caused by poor homogenization of the Triton X-100. gRNA in saliva was also resistant to RNase, unless pre-treated with Triton X-100 (**Fig 2C**), presumably because it is packaged in virions and in EVs as previously suggested [21,22]. Our assays indicated that salivary sfRNA is present in detergent-sensitive structures that protect it from nuclease digestion.

To test whether the aforementioned detergent-sensitive structures were ribonucleoprotein complexes, such as those identified in honeybee jelly [20], we performed sequential Proteinase K (PK) and RNase A/T1 treatments in saliva samples. To prevent PK from digesting the nucleases, we inhibited the former with phenylmethylsulfonyl fluoride (PMSF), which was dissolved in DMSO (see Fig 2 legend). *In vitro*-transcribed sfRNA1 added to uninfected saliva was digested by RNase regardless of PK treatment (**Fig 2D**). In infected saliva, we noted partial digestion of sfRNA by RNase in the absence of PK treatment and this could be attributed to DMSO, which was used to control for PMSF (compare **Fig 2B and 2E**) and is known to destabilize membranes [23]. Contrary to sfRNA, gRNA was not as sensitive to RNase regardless of treatment (**Fig 2F**), which is not consistent with protection by proteins. Moreover, our data suggest that a fraction of the gRNA was packaged in a DMSO-resistant compartment, which is expected for DENV virions [24], whereas sfRNA was packaged in a more DMSO-sensitive compartment.

### sfRNA is present in salivary extracellular vesicles

Given that sfRNA is localized to detergent sensitive compartments, we posited that sfRNA was likely within salivary extracellular vesicles (EVs). Given the paucity of reagents available to biochemically identify mosquito EVs we imaged mosquito saliva using transmission electron microscopy (TEM) to morphologically identify EVs. Uninfected and infected mosquito saliva contained spherical structures surrounded by membranes, which resemble EVs observed by TEM in tick saliva [25]. Diameters of observed EV-like particles ranged from ~100–800nm in uninfected saliva and from ~100–500nm in infected saliva (**S6 Fig**). We conclude that mosquito saliva, both uninfected and DENV2 infected, contains EV-like particles.

**Fig 3:**
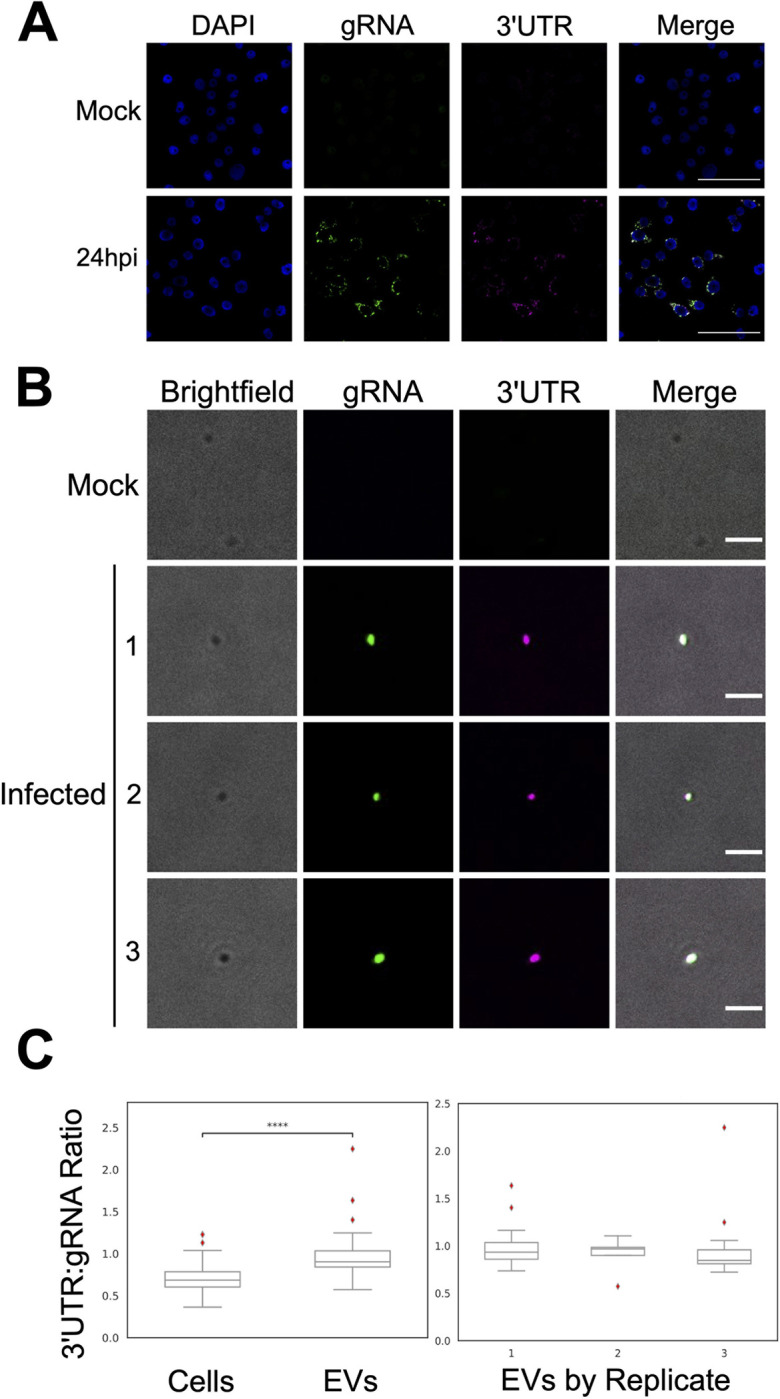
Mosquito salivary EVs are enriched in DENV2 3’UTR RNA, relative to infected cells. **(A)** Representative images of nuclei (blue), DENV2 gRNA (green), and DENV2 3’UTR (magenta) from C6/36 cells 24 h.p.i. with DENV2 NGC at an MOI of 0.3. Scale bar, 50 μm. **(B)** Representative images of EVs imaged in brightfield (grey), DENV2 gRNA (green), and DENV2 3’UTR (magenta) from *Ae*. *aegypti* saliva collected 10 days post inoculation with DENV2 NGC. Scale bar, 2 μm. **(A & B)** RNA-FISH of DENV2 gRNA and 3’UTR labelled with Oligopaints probes. **(C)** Ratios of DENV2 3’UTR: gRNA fluorescence signals as detected via RNA-FISH in DENV2 NGC infected C6/36 cells and EVs from *Ae*. *aegypti* saliva (left). Ratios of DENV2 3’UTR: gRNA fluorescence signals as detected via RNA-FISH on EVs from *Ae*. *aegypti* saliva from the three independent labelling experiments (right). ****, p ≤ 10^−4^ as determined by a two-sided Mann-Whitney U test. Exact p-value = 1.79 x 10^−12^. N_Cells_ = 102; N_EVs_ = 35.

We used Oligopaints RNA fluorescence in situ hybridization (RNA-FISH) probes [26,27] to determine whether viral RNAs in DENV2-infected saliva were found within EV-like particles. Nucleotides 1–10,293 of the DENV2 NGC genome were detected by 240 oligos barcoded as gRNA, while nucleotides 10,294–10,717 were detected by 7 oligos barcoded as 3’UTR, which also detect sfRNA (**S7 Fig**). To test whether our RNA-FISH probes were specifically detecting viral RNAs, we compared RNA-FISH of mock infected and DENV2 infected C6/36 cells and as expected observed signal only in DENV2-infected cells (**Fig 3A**). DENV2-infected cells had the canonical perinuclear staining of viral RNA, with frequent co-localization of gRNA and 3’UTR fluorescent signals (**Fig 3A**). The same result was obtained when the fluorophores used to detect gRNA and 3’UTR probes were swapped (**S8A Fig**). Evidence for labelling specificity was provided by exclusive detection of gRNA probes at very early times post infection (2 hpi) of C6/36 cells, when one expects sfRNA to be undetectable (**S9 Fig**). To further test the specificity of our FISH probes, we transfected an *in vitro*-transcribed DENV2 PR6452 3’UTR RNA into C6/36 cells two hours prior to RNA-FISH labelling. Though there is a 5-nucleotide mismatch between the DENV2 NGC and PR6452 3’UTR sequences [11], imaging of transfected C6/36 cells demonstrated labelling with the 3’UTR but not gRNA probes (**S9 Fig**). These experiments show that the gRNA barcoded RNA-FISH probes did not detect 3’UTR viral sequences and *vice versa*.

Since the Oligopaints RNA-FISH probes were shown to be specific we used them to image uninfected and DENV2-infected mosquito saliva (**Fig 3B**). In uninfected saliva we observed many EVs and as expected the majority of these did not label with the Oligopaints probes (**Fig 3B**). We detected two RNA-FISH staining events in EVs of uninfected saliva and these signals were noted to be non-specific since they associated with the ATTO 565 fluorophore whether used to detect the 3’UTR or gRNA sequences (**S10 Fig**). In DENV2-infected saliva we observed many EVs labeled with both gRNA and 3’UTR probes (**Figs 3B and S11**). EVs positive for 3’UTR appeared to be medium/large EVs [28] (See discussion on limitations of these conclusions below and in **S11 Fig**).

DENV2-infected salivary EVs are enriched in 3’UTR fluorescent labelling compared to C6/36 cells since we observed a median 3’UTR:gRNA fluorescence ratio of 0.685 (± 0.166 SD) in C6/36 cells, and 0.904 (± 0.294 SD) in saliva EVs (**Fig 3C**; p = 1.791 x 10^−12^ with a two-sided Mann-Whitney U test). To ensure that the high ratio of 3’UTR:gRNA labelling observed in EVs was not an artifact of the fluorophores used to visualize hybridized RNA-FISH oligos, we repeated the RNA-FISH experiments with swapped secondary fluorophore conjugated oligos (**S8B Fig**) and again observed enrichment of 3’UTR:gRNA labelling ratios in salivary EVs, which had a median 3’UTR:gRNA fluorescence ratio of 1.512 (± 0.766), as compared to cells, which had a median 3’UTR:gRNA fluorescence ratio of 0.619 (± 0.513) (**S8C Fig**). Overall, our Oligopaints RNA FISH results demonstrate that in DENV2 infected saliva samples, EV-like structures are enriched in DENV2 3’UTR sequences. This enrichment is consistent with the high sfRNA:gRNA ratios detected by RT-qPCR above (**Figs 1 and 2**).

### High sfRNA levels are associated with increased saliva infectivity

While there is ample evidence that sfRNA has potent anti-immune activities [9], we tested how sfRNA levels influenced saliva-mediated infections of human cells. To do this we took advantage of our observations that salivary sfRNA levels varied between independent mosquito infections. We collected pools of saliva from mosquitoes infected with DENV2 strains PR6452, PR1940 and PR9963, and identified those that had similar levels of gRNA copies but substantially different sfRNA levels and therefore had either low or high sfRNA:gRNA ratios. Since sfRNA:gRNA vary significantly between individual mosquitoes we worked with pools of saliva obtained from 40 mosquitoes infected with the same DENV2 strain. By working with saliva pools, we assumed that the impact of any distinct saliva was homogenized between the conditions. In four independent experiments, one with PR6452, two with PR1940 and one with PR9963, we compared infection of human Huh-7 cells with saliva pools with low or high sfRNA:gRNA ratios (**Table 1**). For each comparison between low and high sfRNA: gRNA ratio saliva pools, we infected Huh-7 cells with the equivalent number of viral genomes in the same total volume of saliva, which is known to impact infectivity [2]. To achieve this, we diluted infected saliva samples with saliva from uninfected mosquitoes as described in Table 1.

**Table 1:** Higher sfRNA:gRNA ratio in mosquito saliva increases DENV2 infection of human cells. The experiment number, identity of the virus strain, concentration of gRNA and sfRNA, and sfRNA:gRNA ratio for pools of salivas collected from DENV2 infected mosquitoes are shown in the first five columns. The total volume of salivary inoculum (volume of infected saliva (inf.) + volume of uninfected saliva (uninf.)) used to infect Huh-7 cells and the gRNA copies added per well of Huh-7 cells are indicated in columns six and seven. The geometric mean, lower and upper 95% CI of dsRNA foci per cell at 2 days post infection are in column eight.

Exp.	DENV2 strain	gRNA (copies/ μl)	sfRNA (copies/ μl)	sfRNA: gRNA ratio	Total volume of saliva added per well of Huh-7 cells. (μl)	gRNA inoculum (copies/ well)	dsRNA foci per cell, geometric mean (lower and upper 95% CI)	p-value
1	PR6452	8 x 10^2^	7.2 x 10^3^	9	130 (130 inf. + 0 uninf.)	1 x 10^5^	34.7 (26.2; 46.0)	0.01
	PR6452	9 x 10^2^	4.2 x 10^4^	46.7	130 (115 inf. + 15 uninf.)	1 x 10^5^	64.1 (45.8; 89.8)	
2	PR1940 (#1)	4.2 x10^3^	2.9 x 10^3^	6.7	185 (65.5 inf. + 119.5 uninf.)	2.8 x 10^5^	48.5 (34.7; 67.9)	0.03
	PR1940 (#1)	1.5 x 10^3^	4.6 x 10^4^	30.2	185 (185 inf. + 0 uninf.)	2.8 x 10^5^	78.8 (63.2; 98.3)	
3	PR1940 (#2)	4 x 10^3^	2.2 x 10^3^	5.6	100 (65 inf. + 35 uninf.)	2.6 x 10^5^	49.2 (23.6; 102.2)	0.27
	PR1940 (#2)	2.6 x 10^3^	4.3 x 10^4^	16.6	100 (100 inf. + 0 uninf.)	2.6 x 10^5^	91.6 (53.2; 157.7)	
4	PR9963	3.4 x 10^2^	6.4 x 10^3^	18.8	110 (83 inf. +27 uninf.)	2.8 x 10^4^	51.0 (30.1; 86.5)	0.94
	PR9963	2.6 x 10^2^	5.4 x 10^4^	208	110 (110 inf. + 0 uninf.)	2.8 x 10^4^	54.7 (33.8; 88.6)	

**Fig 4:**
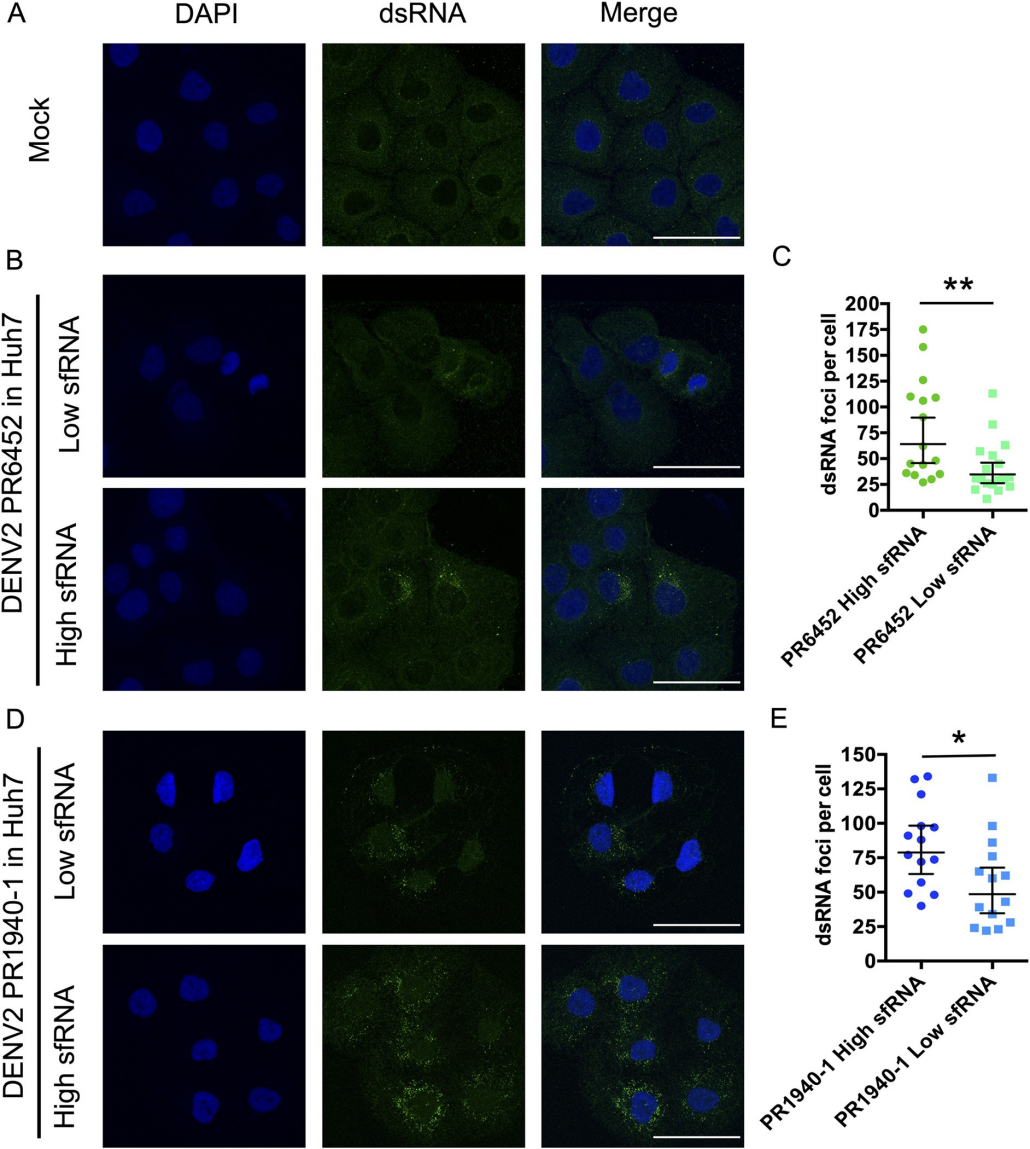
Higher sfRNA:gRNA ratio in mosquito saliva increases DENV2 infection. (**A**) Representative pictures of nucleus (DAPI, blue) and dsRNA (green) from Huh-7 cells supplemented with uninfected saliva. (**B-E**) Representative pictures of nucleus and dsRNA from (**B, D**) and number of dsRNA foci per (**C, E**) Huh-7 cell supplemented with saliva infected with DENV2 PR6452 or PR1940 containing either high or low sfRNA:gRNA ratio. (B, D) Scale bar, 50 μm. (**C, E**) Each point indicates dsRNA foci in one cell. Bars show geometric means ± 95% C.I. *, p-value < 0.05; **, p-value < 0.01 as determined using a Mann-Whitney U test.

As a measure of infection, we quantified foci of viral genome replication detected by immunofluorescence with an anti-dsRNA antibody (**S12 Fig**). We validated that the anti-dsRNA antibody did not detect *in vitro*-transcribed sfRNA transfected into Huh-7 cells (**S12 Fig**). The geometric mean of replication foci per cell was 34.7 (95% CI; lower = 26.2; upper = 46.0) when cells were inoculated with PR6452-infected saliva with a lower sfRNA:gRNA ratio versus 64.1 (95% CI; lower = 45.8; upper = 89.8) for saliva with a higher ratio (**Table 1 and Fig 4B and 4C**). Very similar results were obtained with PR1940-infected saliva pools. In repeat 1, lower sfRNA:gRNA ratio resulted in a geometric mean of 48.5 (95% CI; lower = 34.7; upper = 67.9) foci per cell, whereas higher ratio produced 78.8 (95% CI; lower = 63.2; upper = 98.3) foci per cell (**Table 1 and Fig 4D and 4E**). In repeat 2, lower vs higher ratio resulted in 49.2 (95% CI; lower = 23.6; upper = 102.2) vs 91.6 (95% CI; lower = 53.2; upper = 157.7) foci per cell respectively (**Table 1 and S13 Fig**). This effect was not observed with PR9963-infected saliva pools (**Table 1 and S13 Fig**), perhaps because these saliva samples had 5-10-fold lower viral titers than PR6452 and PR1940. In these experiments we used the percentage of infected Huh-7 cells as a second measure of infectivity and noted that it was higher when cells were exposed to infected saliva samples that had high sfRNA;gRNA ratios (**S14 Fig**).

To determine the impact of salivary sfRNA on cells found at the bite site, we repeated the saliva infection with primary neonatal human dermal fibroblasts (NHDF) (**S1 Table**). Since infection of NHDFs is much less efficient than infection of Huh-7 cells, we quantified intracellular gRNA at 72h post-infection (**S15 Fig**). Whereas we did not detect DENV genomes in cells infected with lower ratios of sfRNA:gRNA, higher ratios resulted in detection of DENV gRNA in NHDF (**S16 Fig**), which suggests that salivary sfRNA enhances infection of human cells found at the bite site. Based on the results above, we propose that high levels of sfRNA in mosquito saliva enhance DENV2 infection in human cells.

### *In vitro*-transcribed 3’UTR RNA enhances DENV2 infectivity and inhibits interferon induction and signaling in human cells

**Fig 5:**
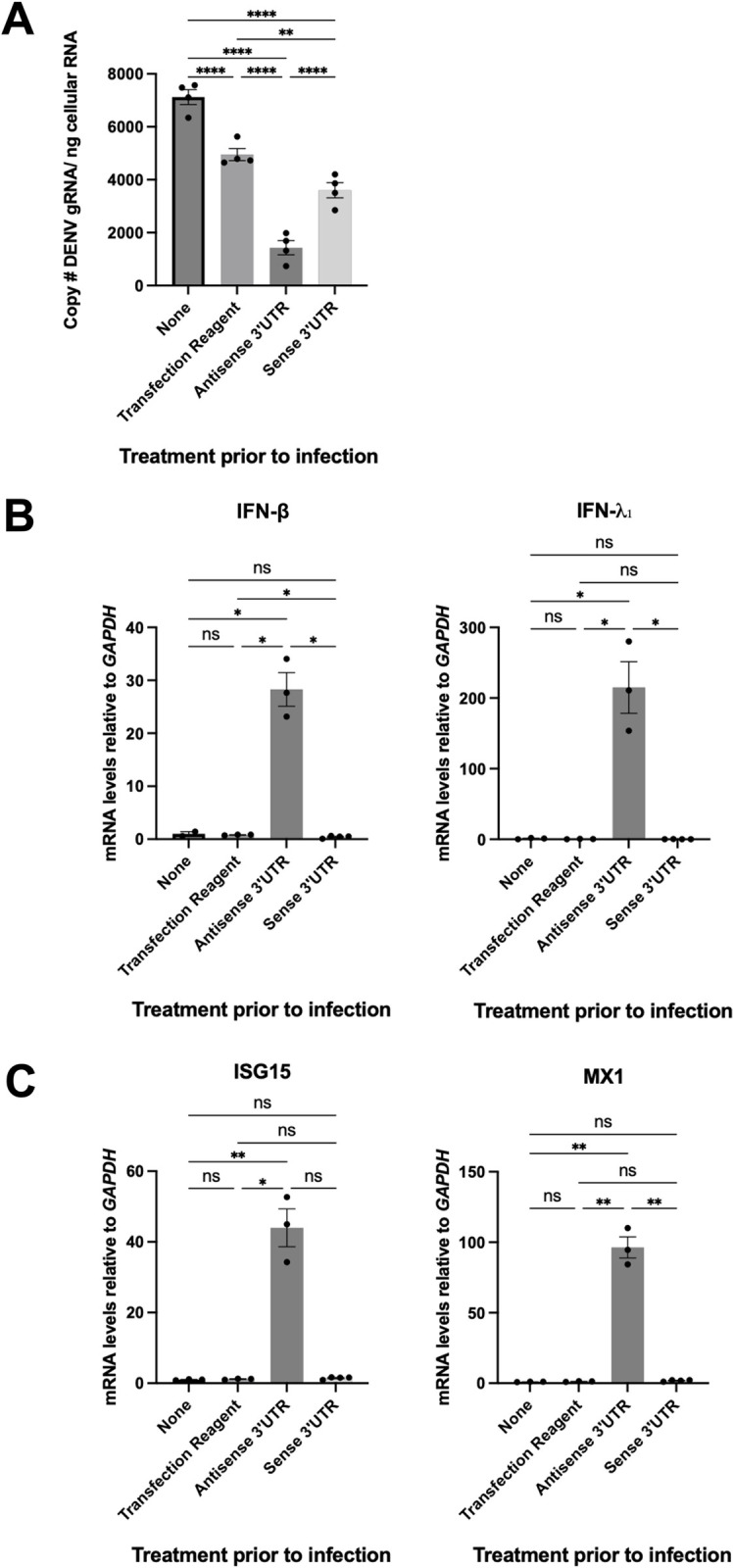
DENV2 3’UTR enhances DENV2 gRNA viral levels while inhibiting innate immunity. Huh-7 cells were either regularly infected (non-treated), or 2 hours prior to infection transfected with sense DENV2 3’UTR, antisense DENV2 3’UTR or lipofectamine alone. 17 hours post infection total cellular RNA was extracted and **(A)** Absolute copy number of intracellular DENV2 gRNA was measured by qRT-PCR. **, p-value ≤ 0.005, ****, p-value ≤ 0.0001 as determined by a Fisher’s LSD test. **(B)** Relative mRNA transcript levels of *IFN-β* and *IFN-λ*_*1*_ measured at 17 hours post infection, normalized first to *GAPDH* mRNA levels, and then to regular infection (“none” treatment post infection). *, p-value ≤ 0.05, as determined by unpaired t-test with Welch’s correction. **(C)** Relative mRNA transcript levels of interferon stimulated genes, *ISG15* and *MX-1* measured at 17 hours post-infection, normalized first to *GAPDH* mRNA levels, and then to regular infection (“none” treatment post infection). *, p-value≤0.05, **, p-value ≤ 0.005. For *ISG15*, p-value was determined by a Dunn’s test. For *MX-1* mRNA levels, p-value was determined by an unpaired t-test with Welch’s correction. Points demonstrate replicate values from one independent experiment. Bars demonstrate arithmetic means ± s.e.m.

To test whether sfRNA is responsible for infection enhancing properties observed in saliva, we mimicked sfRNA delivery by transfecting an *in vitro*-transcribed 5’-monophosphorylated DENV2 3’UTR RNA into Huh-7 cells two hours prior DENV2 infection. To control for the general effects of transfecting a foreign RNA on infection, we transfected a 5’-monophosphorylated antisense 3’UTR RNA. As noted previously transfection of any RNA inhibited infection probably due to activation innate immune and stress pathways (**Fig 5**). Transfection of sense 3’UTR RNA significantly increased the levels DENV2 genomic RNA in infected cells (**Fig 5A**). We did not detect differences in the accumulation of extracellular viral RNAs with any condition tested (**S17 Fig**), but this is not surprising given the short infection times (17 hpi). These data, which are consistent with previously published results, indicate that introduction of DENV2 sfRNA in human cells enhances infectivity.

To determine whether the increased infection following sense 3’UTR transfection correlated with the previously-described sfRNA-mediated immune inhibition [8,11], we monitored IFN induction by quantifying *IFN-β* and *IFN-λ*_*1*_ expression in the same samples. While transfection of antisense 3’UTR increased *IFN-β* and *IFN-λ1* mRNA levels, sense 3’UTR transfection did not **(Fig 5B)**. Additionally, we monitored IFN signaling by quantifying expression of the IFN-stimulated genes (ISGs) *ISG15* and *myxoma resistance 1* (*MX1*). As noted for IFNs, induction of *ISG15* and *MX1* expression by transfection of RNA were markedly repressed by the sense 3’UTR (**Fig 5C**). Together, these data support the hypothesis that sfRNA delivery enhances DENV2 infection by inhibiting IFN induction and subsequent signaling.

#### Limitations of the work

Given the challenges of working with mosquito saliva we felt it was important to include this section where we describe limitations of the work presented.

In order to quantify sfRNA levels in very small volumes of mosquito saliva we used RT-qPCR. We developed a method where primers targeting the coding region detects gRNA and primers targeting the 3’UTR detect both gRNA and sfRNA [8,13]. sfRNA copies are then calculated by subtracting RNA copies quantified with gRNA primers from RNA copies quantified with 3’UTR-targeting primers. This method also detects gRNA decay intermediates and thus overestimates levels of completely processed sfRNAs. Nonetheless, it should be noted that the discovery of salivary sfRNA has been confirmed by multiple methods in three different laboratories.

Our light microscopy-based visualization of EVs, which is diffraction limited, could not resolve objects smaller than 250 nm and therefore observed EV diameters could be smaller than reported (**S11 Fig)**. In order to rigorously test our conclusions, we measured the apparent diameter of 100 nm fluorescent Tetraspeck nanospheres using both 647 nm fluorescence and brightfield and recalculated sfRNA:gRNA signal ratios excluding objects that could be smaller than these microspheres. Importantly, these analyses confirmed that even with application of these exclusion criteria, overall trends of enrichment in 3’UTR FISH labelling of EVs did not change dramatically (**S11 Fig)**.

In the experiments showing infection-enhancing function of salivary sfRNA in primary dermal fibroblasts, we were limited by the fact that these cells do not support high infection level and were sensitive to saliva toxicity, which restricted saliva volume we used as inoculum. These limitations did not permit us to measure infection *per se*.

Finally, although experiments that transfect sfRNA into human cells are fully consistent with our observations with mosquito saliva, it is not certain that transfection of RNA leads to equivalent delivery of this cargo as salivary EVs.

Despite their inherent limitations our complementary methods indicated the presence of sfRNA in salivary EVs and strongly suggested that this ncRNA enhanced DENV2 infectivity.

## Discussion

**Fig 6:**
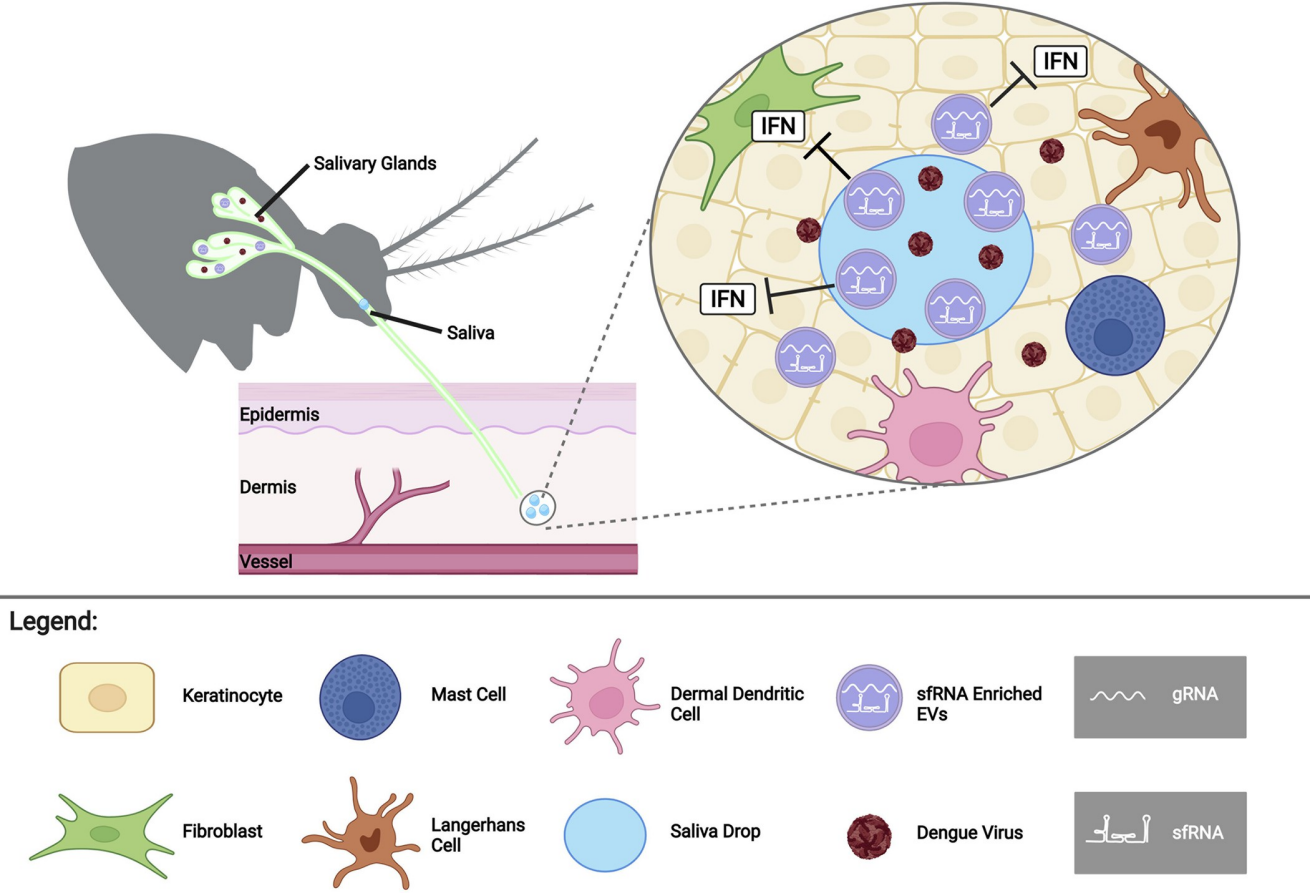
Model: sfRNA in salivary vesicles is transported into cells to enhance infection at the biting site. sfRNA packaged inside extracellular vesicles in infectious mosquito saliva is transferred to human skin cells during biting. sfRNA inhibits the antiviral innate immune response (interferon (IFN) system) to enhance skin cell virus infection. Created with BioRender.com.

Taken together, our results indicate that infection-enhancing sfRNA is secreted in the lumen of detergent-sensitive EVs in mosquito saliva. While it is formally possible that sfRNA is packaged in DENV2 virions in the saliva, as proposed in a recent report studying human EVs [17], we do not favor this conclusion based on the differential DMSO sensitivity observed for gRNA and sfRNA and our previous data [8]. EVs are well known vehicles of coding and noncoding RNAs, including viral RNAs [29–31]. Our observations of EVs in mosquito saliva labelled for DENV2 3’UTR and gRNA, is consistent with previous studies using DENV and other Flaviviridae [21,32–35]. In fact, EVs derived from DENV infected mosquito cells have been isolated, characterized and shown to contain DENV gRNA fragments [21,22]. Furthermore, EVs derived from arthropod cells in culture have been suggested to transmit viral genomes to human keratinocyte-like HaCaT cells [21,33]. Based on these considerations, we propose that sfRNA in saliva is present in EVs, which can deliver their cargo to cells in the same location and at the same time as virus is deposited in the bite site (**Fig 6**).

sfRNA has been shown to have potent anti-immune function in human cells [9,36] which is consistent with data here that show that transfection of a sfRNA mimic prevents RNA-induced IFN response. Based on these data and on our observation that higher levels of sfRNA in saliva correlate with enhanced DENV2 infectivity, we posit that sfRNA delivered to human skin cells during biting is likely to dampen the local innate immune response and give the virus an advantage that favors mosquito transmission. The role of EVs in the battle between pathogens and hosts is not unprecedented. The nematode *Heligmosomoides polygyrus* uses EVs to deliver miRNAs and other transactivators to suppress Type 2 innate immunity [37]. Similarly, hosts can transfer small RNAs to silence virulence genes of pathogens [38]. In this report, we reveal another level of complexity and propose that DENV2 harnesses the biology of its invertebrate vector to increase transmission to the human host.

## Materials and methods

### Cell lines, virus, and mosquitoes

*Aedes albopictus* C6/36 (CRL-1660) and baby hamster kidney BHK-21 (CCL-10) cell lines from ATCC were grown in RPMI media (ThermoFisher Scientific), supplemented with 10% heat-inactivated fetal bovine serum (FBS) (ThermoFisher Scientific) and 1% Penicillin-Streptomycin (ThermoFisher Scientific) at 28°C and 37°C, respectively, in 5% CO_2_. Authentication of C6/36 cells is in progress. Huh-7 cell line (clone JTC-39) was obtained from the Japanese Health Sciences Foundation, Osaka and was maintained in Dulbecco’s modified Eagle medium (DMEM, ThermoFisher Scientific), supplemented with 10% FBS, 1% non -essential amino acids, and 1% Penicillin-Streptomycin at 37°C in 5% CO_2_. Huh-7 cells were authenticated using nine STR loci that had 100% match to the reference (carried out in the UTMB genomics core). Neonatal Human Dermal Fibroblasts (NHDF) (CC-2509, Lonza) were grown at 37°C with 5% CO_2_ in Fibroblast Growth Basal Medium (FBM, Lonza) supplemented with Fibroblast Growth Medium-2 bulletkit (FGM-2, Lonza) and 2% FBS (Gibco). NHDFs are provided with a certificate of analysis from Lonza. All cell lines used were tested for mycoplasma contamination and were shown to be negative. None of the cell lines used are listed among commonly misidentified cell lines by the International Cell Line Authentication Committee.

Dengue virus 2 (DENV2) New Guinea C (NGC) strain was obtained from ATCC (VR-1584) and from the World Reference Center for Emerging Viruses and Arboviruses (WRCREVA) at UTMB. Low-passage stocks of DENV2 PR1940, PR6452, and PR9963 strains [11] were gifts from the Dengue Branch of the Centers for Disease Control and Prevention, San Juan, Puerto Rico, and were obtained from Prof. Eng Eong Ooi’s laboratory at Duke-NUS medical School. DENV2 strains were propagated in C6/36, titrated using plaque assay with BHK-21 cells and stored at -80°C.

The *Aedes aegypti* colony (SGP) from Singapore was established and reared in Duke-NUS insectary as detailed previously [13] (data in **Figs 1, 2 and 4**). The *Ae*. *aegypti* Rockefeller (ROCK) colony was reared using standard methods [39] by the UTMB Insectary Services Core (data in **Figs 1B and 3B**). The *Ae*. *aegypti* colony (BORA) from Bora-Bora was reared at the VectoPole insectary in MIVEGEC as detailed below (data in **S16 Fig**). Eggs were hatched in deionized water and larvae were fed grinded fish food (Tertramin) at 26°C with 12h:12h light:dark cycle until pupation. Adults were maintained in cages (Bioquip) at 28°C, 70% relative humidity with 14h:10h light:dark cycle and provided with 10% sugar solution.

### Mosquito oral infection (Figs 1, 2 and 4)

Three- to five-day-old SGP female mosquitoes were starved for 17 h and offered a blood meal containing 40% volume of washed erythrocytes from specific-pathogen-free pig’s blood (PWG Genetics), 5% of 10 mM ATP (ThermoFisher Scientific), 5% human serum (SigmaAldrich), 50% of RPMI media and 10^7^ plaque forming unit (pfu) per ml in all experiments. NGC from ATCC, PR1940, PR6452, and PR9963 strains were used. Blood meal titer was validated using plaque assay with BHK-21 cells. Mosquitoes were exposed to the blood meal for 1.5h and fully engorged females were selected and maintained with water and 10% sugar solution until analysis at 10 or 15 days post blood meal with NGC or PR strains, respectively.

### Mosquito inoculation (Figs 1B, 3B and S16)

For **Fig 3B**, three- to five-day-old female ROCK mosquitoes were cold-anesthetized and inoculated with 180 pfu of DENV2 NGC from WRCEVA (UTMB). For **S16 Fig**, three- to five-day-old female BORA mosquitoes were cold-anesthetized and inoculated with 50 pfu of DENV2 NGC from ATCC. Inoculation was made using a Nanoject II (Drummond scientific company). Control mosquitoes were inoculated with the same volume of PBS. Following inoculation, mosquitoes were maintained for 10 (**Figs 1B and 3B**) or 7 (**S16 Fig**) days with *ad libitum* access to 10% sucrose solution.

### Mosquito saliva collection (Figs 1–4)

Starved mosquitoes were immobilized by cutting wings and legs. Mosquito proboscises were individually inserted into 20 μl tips containing 10 μl of pre-warmed media with 0.5 mM ATP for 30 min. For **Figs 1 and 2**, the media was RPMI supplemented with 0.01% Rhodamine to identify mosquitoes that ingested Rhodamine, hence salivated. For **Fig 3B**, the media was RPMI. For **Fig 4**, the media was DMEM. For **S16 Fig** the media was FBM media.

### Northern blots (Fig 1A)

Northern blots used the NorthernMax Kit (Ambion) with modifications to manufacturer’s protocol as described [13,16,40]. Total RNA from 50 orally infected mosquitoes was extracted using RNAzol RT (Molecular Research Center) and separated on a denaturing gel with 5% acrylamide (Bio-Rad) and 8 M Urea (1^st^ Base). Biotinylated single-stranded RNA ladder (Kerafast) was also loaded on the gel. RNA was transferred onto a nylon membrane (Hybond-N; Biodyne B) using Trans-Blot Turbo (Bio-Rad) at 25 V for 30 min. The membrane was UV-crosslinked, pre-hybridized and hybridized with a biotin-16-dUTP (Roche) labeled dsDNA probe including the whole 3’UTR and generated using previously detailed primers [8]. After washes and blocking, the membrane was stained with IRDYE 800cw streptavidin (LI-COR). Pictures were taken with Odyssey CLx imaging system (LI-COR).

### Northern blots (Fig 1B)

Northern blots used the NorthernMax Kit (Ambion) with modifications to manufacturer’s protocol as described with the following modifications below [13,16,40]. Total RNA from the saliva of 30 DENV2 and mock (PBS) inoculated mosquitoes was extracted using Trizol LS (Thermo Fisher Scientific) according to the manufacturer’s protocol with the following modifications: RNA was precipitated with 1uL of GlycoBlue reagent (Thermo Fisher Scientific) at -20°C for 2h. Total extracted RNA was mixed with 2X RNA loading dye (New England Biolabs) and separated on a 6% acrylamide TBE-Urea gels (Thermo Fisher Scientific) at 110V for 2.5h with 1X TBE. Biotinylated single-stranded RNA ladder (Kerafast) was also loaded on the gel. As a control to visualize sfRNA we used total cellular RNA from uninfected Huh-7 cells, along with total RNA from Huh-7 cells infected with DENV2 NGC, with an MOI of 2, at 48 hours post infection (**S18 Fig**). The gel was transferred to a Biodyne B nylon membrane (Thermo Fisher Scientific) using the XCell II Blot module (Thermo Fisher Scientific) at 15V for 1.5h in 0.5X TBE. The membrane was UV crosslinked, and pre-hybridized as previously described. To visualize sfRNA, 1ug of biotin 16-dUTP (Roche) labelled dsDNA probe was used, generated using previously detailed primers [8]. The blot was hybridized at 78°C overnight. Following overnight hybridization, two low-stringency washes were completed at room temperature for 10 min each. All other washes were according to the NorthernMax (Ambion) protocol, and as previously detailed. Lastly, the membrane was stained with IRDYE 800cw streptavidin (LI-COR) and imaged with the Odyssey CLx imaging system (LI-COR).

### Absolute quantification of gRNA and sfRNA1 copies by real-time RT-qPCR (Figs 1, 2, and 4)

Orally infected mosquitoes were used. Salivary glands were homogenized with 1.0 mm silica beads (BioSpec) using mini-beadbeater-96 (BioSpec) in 350 μl of TRK lysis buffer and saliva-containing mixtures were lysed in 350 μl TRK lysis buffer. Total RNA was extracted with E.Z.N.A. Total RNA kit I (Omega Bio-Tek). gRNA and 3’UTR/sfRNA1 were quantified in absolute numbers using one-step RT-qPCR (**S2 Table**) and standard curves as detailed [13].

To calculate the limit of detection (LoD), gRNA and 3’UTR/sfRNA1 fragments encompassing the qPCR targets and generated for the standard curves were 10- or 2-fold serially diluted from 1.2 x 10^7^ to 1.9 copies for gRNA and from 6 x 10^8^ to 117 copies for sfRNA1. Four technical repeats per dilution of three independent repeats were quantified using one-step RT-qPCR. Fractions of positive replicates per copies of gRNA or 3’UTR/ sfRNA1 were plotted against a sigmoid curve to identify LoD at 95% confidence [41].

sfRNA1 copy number was calculated by subtracting the number of sfRNA1 and 3’UTR fragments (estimated using the 3’UTR/sfRNA1 primers) to the number of gRNA fragments (estimated using the envelope primers). For infected samples that contained detectable amount of sfRNA, sfRNA:gRNA ratio was calculated by dividing the number of sfRNA over the number of gRNA. gRNA detection rate corresponded to the number of samples with detectable amount of gRNA over the total number of analyzed samples. sfRNA detection rate corresponded to the number of samples with detectable amount of sfRNA over the number of samples with detectable amount of gRNA.

### sfRNA sequencing (Fig 1)

Total RNA from 50 whole mosquitoes or 10 saliva samples collected after oral infection was extracted using RNAzol RT. sfRNA fragments were circularized using T4 RNA ligase 1 (New England Biolabs) at 4°C overnight [16], reverse transcribed using SuperScript III (ThermoFisher Scientific) with primer 5’-GCTGTTTTTTGTTTCGGG-3’ located at nucleotides 10621–10604 of DENV2 NGC genome and amplified using forward primer 5’-AAAATGGAATGGTGCTGTTG-3’ (nucleotides 10690–10709 from NGC genome) and the primer used for reverse transcription [16]. PCR products were gel-separated and the expected-size bands were cloned with Topo TA kit (ThermoFisher Scientific) before sequencing (1^st^ Base).

### RNase resistance assay after detergent or proteinase treatments (Fig 2)

Twenty saliva samples collected from NGC orally infected or uninfected mosquitoes were pooled and divided into four equal volume fractions. Each subset was used to test one of the four combinations of RNase with either Triton X-100 or proteinase K (PK) treatments. *In vitro*-transcribed sfRNA1 generated for standard curve (see above) was added to each uninfected saliva subgroup. Samples were treated with 0.1% Triton X-100 (Sigma) at room temperature for 30 min before adding 3 μl of RNase A/T1 (ThermoFisher Scientific) at 37°C for 30 min. To disperse Triton X-100 solution while maintaining EV integrity, we gently pipetted up and down for ten times for the treatment mixture. Samples were treated with 0.25 μg of PK (ThermoFisher Scientific) at room temperature for 30 min and with 2 mM phenylmethylsulfonyl fluoride (PMSF) (Sigma) at room temperature for 10 min to inhibit PK before adding 3 μl of RNase A/T1 at 37°C for 30 min. As PMSF was dissolved in DMSO (Sigma), 1% DMSO was added as control when PMSF was not added. RNA was extracted using QIAamp viral RNA mini kit (Qiagen) and sfRNA and gRNA were quantified.

### TEM of *Ae*. *aegypti* mosquito saliva (S6 Fig)

Methods were modified from Théry et al. (2006) [42]. 10uL of pooled saliva from uninfected (mock) and DENV2 infected mosquitoes was directly absorbed onto formvar copper grids (Electron Microscopy Sciences) for 10 min and excess saliva blotted away. Next, grids were fixed by floating on a mixture of 2% glutaraldehyde (Electron Microscopy Sciences) and 2% paraformaldehyde (Electron Microscopy Sciences) for 10 min and excess fixative blotted away. Grids were then floated on a solution of uranyl acetate (Electron Microscopy Services) and 0.15M oxalic acid (Sigma-Aldrich) at a 1:1 ratio for 5 min and excess uranyl oxalate blotted away. Lastly, grids were embedded by floating on a solution containing 2% methyl cellulose (Sigma-Aldrich, 25 centripose, M-6385) and 4% uranyl acetate (Electron Microscopy Services) at a ratio of 9:1 for 10 min on ice. Grids were examined in a JEOL JEM-1400 (USA) transmission electron microscope at 80 kV. Images were acquired on a bottom-mounted CCD camera Orius SC2001 (Gatan, Pleasanton, CA).

### Oligopaints Library Design (Fig 3)

Using the Oligominer pipeline [27], the Oligopaints RNA FISH probe library was designed to bind DENV2 NGC genome. Briefly, the blockParse script was used to select oligos between 35–41 nt long with a T_m_ of 42–47°C. The selected oligos were aligned to the DENV2 NGC, *Homo sapiens* (hg38, GenBank Accession #: GCA_000001405.28) and *Ae*. *aegypti* (AaegL5.0, GenBank Accession #: GCA_002204515.1) genomes using bowtie2 [43]. Oligos aligning with *H*. *sapiens* or *Ae*. *aegypti* genomes or having target site at 42°C in these genomes were identified using the outputClean script and manually removed from the library [27]. Candidate oligos were further filtered using the kmerFilter script from JellyFish algorithm [44] with the following parameters: ‘-k 5 -m 18’ and oligos with 18-mers occurring more than five times in the DENV2 NGC genome were removed. Lastly, oligos binding on reverse complements to the DENV2 genome were generated with probeRC script [27]. Overall, the library consists of 240 oligos with an average density of 22 oligos/kb of DENV2 genome.

Main- and Back street sequences were appended to the 5’ and 3’ end (respectively) of the DENV2 Oligopaints library using the OligoLego appending tool [45]. 233 oligos binding nucleotides 1–10,293 on the DENV2 NGC genome were barcoded as gRNA, while 7 oligos binding nucleotides 10,294–10,717 were barcoded as 3’UTR. Sequences of MainStreet barcodes, bridge oligonucleotides, and fluorophore conjugated secondary oligonucleotides are detailed in **S7 Fig**.

### APTES Coating of ibidi cover glass chamber slides (Fig 3)

μ-Slide 8 well high glass bottom slides (ibidi) were washed with 200 μL of acetone (Fisher Scientific), treated with 3M KOH (Sigma-Aldrich) and sonicated for 1 hour. After washes with water, each well was incubated with 200 μL of 2% (vol/vol) 3-Aminoporpyltioethoxysilane (APTES) (Sigma-Aldrich) in dry acetone for 30 sec. Slide chambers were washed twice with 400 μL of water and dried for 3 hours at 37°C.

### Oligopaints RNA FISH staining (Fig 3)

2 x 10^5^ C6/36 cells were seeded into μ-slides and infected with DENV2 NGC from WRCEVA at MOI of 0.3 for 1 hour. Control cells were mock-infected with PBS. After infection, cells were maintained in RPMI media supplemented with 5% FBS (GenClone), 0.01 M HEPES (pH 7.4) (Gibco), and 1% Penicilin/Streptomicin (Gibco) for 2 or 24 hours. Following incubation, media was removed, and cells were rinsed twice with 1X PBS. Pools of saliva collected from inoculated mosquitoes were dried on APTES-coated μ-slides for 20 min. Cells and saliva pools were fixed with 4% paraformaldehyde (Sigma-Aldrich) for 10 min, permeabilized with 0.5% Triton X-100 for 15 min (Sigma-Aldrich), blocked with 5% BSA (Sigma-Aldrich) for 1 hour, washed with 1% Tween-20 (Sigma-Aldrich) and rinsed with 1% Tween-20 in 2X saline-sodium citrate buffer (SSC) (Sigma Aldrich). The primary hybridization solution consisting of 1% Tween-20 in 2X SSC, 10% dextran sulfate (Sigma-Aldrich), 50% formamide (Fisher Scientific), 200 μg/μL of sheared salmon sperm DNA (Stratagene), 100 μg/μL of yeast tRNA (Sigma), and 4μM of the Oligopaints DENV2 3’UTR/gRNA FISH probe library was added and samples were incubated in a 60°C water-bath for 3 min. Samples were washed several times with pre-warmed (60°C) 1% Tween-20 in 2X SSC, twice with 1% Tween-20 in 2X SSC at RT and once with PBS at RT. The secondary hybridization solution consisting of 2X SSC, 0.03% Tween-20, 30% formamide, 5% dextran sulfate, 200 μg/μL sheared salmon sperm DNA, 100 μg/μL yeast tRNA, 0.5 μM of bridge oligos, 0.5 μM of ATTO 565 and Alexa Fluor 647 conjugated secondary oligos (Integrated DNA Technologies) was added and samples were incubated for 30 min at RT. Samples were washed four times for 5 min with 1% Tween-20 in 2X SSC and 35% formamide. C6/36 samples were placed in an image buffer consisting of 4 mM Protocatechuic acid (PCA), 15 nM protocatechuate 3,4-dioxygenase (PCD) and 1.5 mM 6-Hydroxy-2,5,7,8-tetramethylchroman-2-carboxylic acid (Trolox) as previously described with the addition of 300 nM of 4,6-diamidino-2-phenylindole (DAPI) (Sigma-Aldrich) [46]. Mosquito saliva samples were placed in the same buffer without DAPI.

Confocal imaging was performed on a Leica TCS SP8 STED 3X system (Leica Microsystems, Wetzlar Germany) equipped with a tunable (470–670 nm) pulsed white light laser (Leica WLL) and a UV 405 nm diode laser for excitations at 405 nm, 564 nm and 647 nm. Emission for DAPI, ATTO 565, and Alexa Fluor 647 was monitored in the 413–450 nm, 568–640 nm and 653–710 nm range, respectively using the built-in acousto-optical beam splitter in combination with a prism-based tunable multiband spectral detection of the confocal system. Images were acquired using a Leica HC PL APO CS2 100×/1.4 oil immersion objective.

### *In vitro*-transcription and transfection of DENV2 PR6452 3’UTR (S9 Fig)

DENV2 3’UTR was *in vitro*-transcribed from a pcDNA 3.1-Apt-PR6452-3’UTR vector containing T7 RNA Polymerase promoter sequence, streptomycin/tobramycin RNA aptamer sequence, DENV2 PR6452 3’UTR sequence [11]. Briefly, pcDNA 3.1-Apt-PR-3’UTR was linearized with XbaI (NEB), transcribed with HiScribe T7 RNA synthesis kit (NEB), treated with DNAse I (Thermo Fisher Scientific) and purified with phenol-chloroform extraction. RNA integrity was assessed on denaturing 1% agarose gel.

C6/36 cells were transfected with 1 μg of the 3’UTR RNA using Lipofectamine 3000 (Thermo Fisher Scientific). Two hours post-transfection, cells were rinsed twice with 1X PBS, fixed for 10 min with 4% of paraformaldehyde and processed for RNA-FISH, as described above.

### Quantification of 3’UTR:gRNA ratios from images (Fig 3)

*C6/36 cells*. To segment each cell in the images and include both inner and outer nuclei signal in the analysis we added maximum intensity projections of the 3’UTR probes, gRNA probes and DAPI image stacks. We applied a maximum filter to fill empty areas within each cell followed by a gaussian filter to smooth the images. The resulting images were converted to binary masks and used to identify the area occupied by each individual cell using ImageJ [47]. To guarantee that only infected cells were analyzed, we measured the maximum gRNA integrated density in the pool of mock (uninfected) cells and used this value to discard cells in the infected images. The 3’UTR/sfRNA:gRNA ratios were computed by collapsing the image stacks to maximum intensity projections and by dividing the integrated densities in each segmented cell (sum of the values of the pixels in the selection). This was done with the “segment_and_measure_cells.ijm” ImageJ macro (https://github.com/Garcia-BlancoGroup/Yehetal2022).

In the case of 3’UTR/sfRNA images with nuclear autofluorescence signal (**S8A Fig**), we used the cytoplasmic integrated densities of gRNA and 3’UTR/sfRNA signals for analysis. Using ImageJ, we manually segmented the nuclear area using the DAPI images and emptied the area occupied by the nucleus in both 3’UTR/sfRNA and gRNA image stacks [47]. Once removed, the 3’UTR/sfRNA: gRNA ratios were computed as described above, using the “segment_and_measure_cells.ijm” ImageJ macro (https://github.com/Garcia-BlancoGroup/Yehetal2022).

*Extracellular vesicles (EVs)*. To segment each EV, we identified the best in-focus z-slice in wide-field image stacks and manually segmented the vesicle area using ImageJ [47]. This was done with the “segment_vesicles_best_focus.ijm” ImageJ macro (https://github.com/Garcia-BlancoGroup/Yehetal2022). 3’UTR/sfRNA: gRNA ratio was calculated as for cells using the “measure_vesicles_best_focus.ijm” ImageJ macro (https://github.com/Garcia-BlancoGroup/Yehetal2022).

### RT-PCR of DENV2 from mosquito saliva (Related to S10 Fig)

Total RNA from mosquito saliva and infected C6/36 supernatants was extracted with Trizol LS (Thermo Fisher Scientific). cDNA was synthesized using the high-capacity cDNA Reverse Transcription kit (Thermo Fisher Scientific) and qPCR was performed using SYBR Green PCR Master Mix (Thermo Fisher Scientific) with the StepOne Plus instrument (Applied Biosystems) using primers in **S2 Table**. Samples were loaded onto a 1% agarose gel and stained with ethidium bromide to visualize PCR products.

### Imaging of tetraspeck nanospheres (S11C Fig)

100nm fluorescent Tetraspek nanospheres (Thermo Fisher Scientific) were sonicated for 10 min, diluted 1:5000 in 1X PBS, and vortexed. 200 μl of diluted nanospheres were adhered to μ-Slide 8 well high glass bottom slides for 1 hour. Following adhesion, slides were washed 2X with PBS before imaging. Apparent diameters of the nanospheres were determined using ImageJ [47].

### Infection of human cells by incubation with infected mosquito saliva (Fig 4)

3,000 Huh-7 cells were seeded in each well of a 12-well removable chamber slide (ibidi GmbH) and grown in 200 μl of complete media. Media was removed and cells were incubated with pools of 40 orally infected mosquito salivas for two hours at 37°C. Saliva inoculum was removed and replaced with 200 μl of DMEM supplemented with 2% FBS (See **Table 1**). Two days post infection, cells were fixed with 4% paraformaldehyde for 20 min, permeabilized with 0.1% SDS (Merck) in PBS for 10 min, blocked with 0.1% of BSA in PBS for 40 min, and incubated with 1:500 anti-dsRNA J2 antibody (Sigma-Aldrich) in blocking solution for 1 hour at RT. After two PBS washes, cells were incubated with 1:1000 Alexa Fluor 488-labeled anti-mouse IgG antibody (Invitrogen) in blocking media for 1 hour. After washes, slides were mounted with Prolong gold Antifade Mountant containing DAPI (Invitrogen). Pictures were taken with TCS SP8 STED 3X nanoscope (Leica). dsRNA foci were detected and quantified with ImageJ [47] using an in-house macro (https://github.com/Garcia-BlancoGroup/Yehetal2022) that processed the background at 50 pixels, adjusted the threshold to remove the noise signal, and quantified particle size larger than 0.08 μm^2^.

To test if the anti-dsRNA J2 antibody detected sfRNA, full length sfRNA1 from DENV2 NGC was *in vitro*-produced as detailed above with the primers V1.25: TAA TAC GAC TCA CTA TAG GGG AAG TCA GGT CGG ATT AAG C and V1.26: AGA ACC TGT TGA TTC AAC AGC AC. 5 x 10^5^ Huh-7 cells were either mock-infected, infected with DENV2 NGC from ATCC at MOI of 1 for 1 hour, or transfected with folded sfRNA using TransIT-mRNA (Mirus). Cells were stained at 24 hours post infection or 1 hour post sfRNA transfection as detailed above and observed with a LSM780 confocal microscope (Zeiss). To quantify intracellular sfRNA we washed cells with PBS, collected them in TRK lysis buffer, extracted RNA with E.Z.N.A. Total RNA kit I and quantified sfRNA by RT-qPCR, as detailed above (**S12 Fig**).

### Quantification of dermal fibroblast infection with saliva (S16 Fig)

Pools of 40 saliva samples from inoculated mosquitoes were filtered through 0.22 μm filters (Millex-GV, Millipore) and stored at -80°C until use. 3,000 NHDF cells were grown with 200 μl of fibroblast complete media supplemented with 2% FBS in 12-well removable chamber slide. 24 hours post-seeding, media was removed and cells were incubated with 25 μl of saliva diluted with 25 μl of FBS-free fibroblast media for 1 hour upon gentle rocking. The gRNA copies from the different infected saliva pools were normalized and non-infected saliva was used to adjust to a total volume of 25 μl of saliva. The inoculum was removed and replaced with 200 μl of fibroblast media containing 0.5% FBS. 72 hours post infection, cells were collected in 250 μl of TRK lysis buffer and total RNA was extracted following manufacturer’s instructions (E.Z.N.A Total RNA extraction kit I).

gRNA was quantified with one-step RT-qPCR using iTaq Universal SYBR green one-step kit (Bio-Rad) and primers NS5 D2+: 5’-CTCCCTGAGTGGAGTGGAAG-3’ and NS5 D2-: 5’-ACACGCACCACCTTGTTTTG-3’ for gRNA. The reaction mix contained 300 nM of forward and reverse primers and 2 μl of RNA extract in a total volume of 10 μl. The RT-qPCR assays were performed on AriaMx Real-time PCR System (Agilent) with the following thermal profile: 50°C for 10 min, 95°C for 1 min and 40 cycles of 95°C for 10 sec and 60°C for 15 sec followed by a melting curve analysis. An absolute standard curve for gRNA was generated as detailed previously [13]. We calculated a 95% LoD for gRNA as above [41]. gRNA qPCR target generated for the standard curves were 10- or 2-fold serially diluted from 2,202 to 0.04 copies. Six technical repeats per dilution were quantified using one-step RT-qPCR (**S15 Fig**).

### *In vitro*-transcription and transfection of DENV2 NGC 3’UTR (Fig 5)

DENV2 sense and antisense 3’UTR was *in vitro*-transcribed from a PCR product amplified from pcDNA 3.1-DENV2 NGC 3’UTR vector containing the DENV2 NGC 3’UTR sequence. Briefly, 100ng pcDNA 3.1-DENV2 NGC 3’UTR was used as a template for PCR using the DENV2 3’UTR sense and antisense primers. Sense 3’UTR was amplified using the T7 3’UTR Sense F primer (5’- TAATACGACTCACTATAGGGAAGGCAAAACTAACATGAAAC 3’) and 3’UTR Sense R primer (5’- AGAACCTGTTGATTCAACAG 3’). Antisense 3’UTR was amplified using the T7 3’UTR Antisense F primer (5’- TAATACGACTCACTATAGGGAGAACCTGTTGATTCAACAG-3’) and 3’UTR Antisense R primer (5’- AAGGCAAAACTAACATGAAAC-3’). PCR was performed using the Taq 2X master mix (New England Biolabs) according to the manufacturer’s protocol. PCR products of the sense and antisense 3’UTR amplicons were purified using Nucleospin Gel and PCR Cleanup Mini Kit (Macherey-Nagel). Purified PCR products were *in vitro*-transcribed with HiScribe T7 RNA synthesis kit (NEB), treated with DNAse I (Thermo Fisher Scientific) and purified with phenol-chloroform extraction. RNA integrity and specificity was assessed on denaturing 1% agarose gel and RNAse T1 treatment (Thermo Fisher Scientific). Finally, sense and antisense 3’UTR RNAs were treated with RNA 5´ Polyphosphatase (Lucigen) and cleaned up using RNA Clean and Concentrator kit (Zymo) according to the manufacturer’s protocol.

1x10^5^ Huh-7 cells were grown in a 48 well plate and transfected with 50 ng of sense and antisense 3’UTR RNA using Lipofectamine 3000 (Thermo Fisher Scientific) and topped up with DMEM (Thermo Fisher Scientific) supplemented with 5% FBS. 2 hours post transfection, any remaining media was removed, and cells were infected with DENV2 NGC at an MOI of 0.1 in DMEM (Thermo Fisher Scientific) supplemented with 5% FBS, for 2 hours at 37°C. After infection, any remaining virus inoculum was removed and cells were maintained in 200 μL of DMEM (Thermo Fisher Scientific) supplemented with 10% FBS, 1% Non -Essential Amino acids, and 1% Penicillin-Streptomycin.

### Absolute quantification of DENV2 gRNA copies and relative quantification of mRNA transcripts from 3’UTR transfected Huh-7 cells (Fig 5)

At 17 hours post infection, cellular supernatants were harvested from transfected cells, and spun for 20 minutes at 3000 x *g* to pellet any cellular debris. 140 μL of cleared supernatant was immediately processed for viral RNA extraction using the QIAamp viral RNA Mini Kit (Qiagen) according to the manufacturer’s protocol. Remaining transfected cells were washed 2X with PBS and total RNA was extracted using 140 μL of Trizol and the Direct-zol RNA Purification kit (Zymo) according to the manufacturer’s protocol. Intra- and extracellular RNA was used for cDNA synthesis using the high-capacity cDNA Reverse Transcription kit (Thermo Fisher Scientific). Intracellular cDNA was diluted 1 in 5, while extracellular cDNA was diluted 1 in 25 with water. 2.5 μL of diluted cDNA was used for qPCR using the iTaq Universal SYBR Green Supermix (Bio-Rad) in a 10 μL reaction. The reaction mix contained 400 nM of forward and reverse primers for DENV2 gRNA (**S2 Table**). qPCR was performed on the BioRad CFX384 Real Time System instrument using cycling conditions previously described [8]. Absolute copy number of DENV2 gRNA was determined via a standard curve as previously described **(S19 Fig**) [8].

mRNA transcript levels were quantified via relative qPCR from intracellular cDNA samples described above. 2.5 μL of diluted cDNA was used for qPCR using the iTaq Universal SYBR Green Supermix (Bio-Rad) in a 10 μL reaction. The reaction mix contained 300 nM of forward and reverse primers for IFNs and ISGs listed in **S2 Table**. qPCR was performed on the BioRad CFX384 Real Time System instrument using cycling conditions previously described [8]. *GAPDH* mRNA levels were used for normalization of C_T_ values (**S2 Table**).

### Statistical analysis

Differences in sfRNA:gRNA ratio, log-transformed gRNA and sfRNA copies were tested with unpaired student’s t-tests (**Fig 1**). Multiple comparisons with Fisher’s LSD test were used for resistance assays (**Fig 2**). dsRNA foci and the percentage of dsRNA positive cells were tested with Mann-Whitney U test (**Figs 4, S13 and S14**). Prism v5 (GraphPad) was used for analysis. For RNA-FISH images ***(*Figs 3C and S8C*)***, differences in 3’UTR/sfRNA:gRNA ratio were tested using a two-sided Mann-Whitney U test with R [48]. Differences in apparent EV diameters ***(*S11A–S11C Fig*)*** were tested using a two-sided student’s t-tests with Welch’s corrections for unequal variance test with R [48]. Differences in absolute gRNA copy numbers **(Figs 5 and S17)**. were tested using an uncorrected Fisher’s LSD using Prism v9 (Graphpad). Differences in relative mRNA levels of IFN transcripts and ISGs **(Fig 5)** were tested using an unpaired t-test with Welch’s correction and uncorrected Dunn’s test using Prism v9 (GraphPad).

## Supporting information

S1 Fig (Related to Fig 1). **Limit of detection (LoD) and equation for absolute quantification of DENV2 gRNA.** *In vitro* transcribed RT-qPCR target was serially diluted from 1.2 x 10^7^ to 1.9 copies before quantification by RT-qPCR. (A) Fractions of positive replicates were plotted against gRNA copies. At 95% confidence, LoD of log2 DENV2 gRNA is 7.065, equaling 133 copies. (B) Standard curve for absolute quantification of gRNA copies. (A-B) Points show means ± s.e.m. from 12 technical replicates from 3 independent experiments.
(TIF)

S2 Fig (Related to Fig 1). **Limit of detection (LoD) and equation for absolute quantification of DENV2 3’ UTR/ sfRNA1.** *In vitro* transcribed RT-qPCR target was serially diluted from 6 x 10^8^ to 117 copies before quantification by RT-qPCR. (A) Fractions of positive replicates were plotted against sfRNA copies. At 95% confidence, LoD of log2 DENV2 3’UTR/ sfRNA1 is 14.93, equaling 31,216 copies. (B) Standard curve for absolute quantification of 3’UTR/ sfRNA1 copies. (A-B) Points show means ± s.e.m. from 12 technical replicates from 3 independent experiments.
(TIF)

S3 Fig (Related to Fig 1). **Detection of sfRNA in DENV2 PR strains infected mosquito saliva.** (A) gRNA; (B) sfRNA; and (C) sfRNA: gRNA ratio in DENV2 PR1940, PR6452, and PR9963 infected saliva. Points represent one saliva collected from one mosquito. (A-B) Bars represent geometric means ± 95% C.I. and dashed lines indicate limits of detection which were 133 copies in gRNA (A) and 31,216 copies in 3’ UTR/ sfRNA1 (B). (C) Bars represent arithmetic means ± s.e.m. PR1940 saliva, N = 58; PR6452 saliva, N = 56; PR9963 saliva, N = 52 from two mosquito batches.
(TIF)

S4 Fig (Related to Fig 1). **sfRNA sequences from whole mosquitoes** Whole mosquitoes were collected at 10 days post oral infection with DENV2 NGC strain. sfRNA sequences were obtained by RNA circularization, directed expansion of circularized templates, cloning of expected-size amplicons and sequencing. Out of 27 clones sequenced, we found sequences corresponding to sfRNA1 and sfRNA3, and one corresponding to a decay intermediate (gc370-4).
(TIF)

S5 Fig (Related to Fig 1). **sfRNA sequences from saliva.** Saliva samples were collected at 10 days post oral infection with DENV2 NGC strain. sfRNA sequences were obtained by RNA circularization, directed expansion of circularized templates, cloning of expected-size amplicons and sequencing. Out of 26 clones sequenced, we found four sequences corresponding to sfRNA1 and sfRNA3.
(TIF)

S6 Fig (Related to Fig 3). **Imaging of mosquito salivary EVs using TEM.** Representative EVs observed in pooled *Ae*. *aegypti* saliva collected from uninfected (mock) mosquitoes or from mosquitoes 10 days post inoculation with DENV2 NGC. Four representative panels of observed EVs are shown per group (mock, DENV2 infected). Scale bars, 0.1 μm.
(TIF)

S7 Fig (Related to Fig 3). **Overview of Oligopaints RNA FISH oligo design.** (A) Design of RNA-FISH oligos barcoded for DENV2 NGC gRNA and 3’UTR. Each oligo consists of a genome homology region (35–41 nt, black), with a 5’ main street region (47nt, red,blue) and 3’ back street region (47nt, orange,red). The main and back street regions both contain universal primer sequences (20nt), gRNA/3’UTR specific barcodes (20nt, blue), and 7nt toehold sequences (not shown). The main street region contains a Sec6 barcode (20 nt, blue) spanning nt 1–10,293 of the DENV2 NGC genome, barcoding the gRNA. The main street region also contains a Sec5 barcode (20 nt, blue) spanning across nt 10,294–10,717 of the DENV2 NGC genome, barcoding the 3’UTR. The back street region contains region-specific barcodes not utilized in these experiments (20nt, orange). Following primary hybridization of the RNA-FISH oligos to the DENV2 RNA, bridge oligos (blue, grey) bind to the main street gRNA/3’UTR barcodes, allowing for either ATTO-565 or Alexa Fluor 647 conjugated secondary oligos to bind, labelling DENV2 gRNA/3’UTR with different fluorophores. (B) Conceptual schematic of RNA-FISH probe hybridization to DENV2 RNA. 240 total RNA-FISH oligos were designed to span the entire DENV2 NGC genome. 233 of which bind to nucleotides 1–10,293 of the DENV2 genome, barcoded as gRNA. 7 RNA-FISH probes bind to nucleotides 10,294–10,717 of the DENV2 genome, barcoded as 3’UTR.
(TIF)

S8 Fig (Related to Fig 3). **Mosquito salivary EVs are enriched in DENV2 3’UTR RNA, relative to infected cells, irrespective of fluorophores.** (A) Representative images of nuclei (blue), DENV2 gRNA (magenta) and DENV2 3’UTR (green) from C6/36 cells 24 h.p.i. with DENV2 NGC at an MOI of 0.3. Scale bar, 50 μm. Note non-specific nuclear signal observed with 3’UTR probes. (B) Representative images of EVs imaged in brightfield (grey), DENV2 gRNA (magenta), and DENV2 3’UTR (green)from *Ae*. *aegypti* saliva collected 10 days post inoculation with DENV2 NGC. Scale bar, 2 μm. (A, B) DENV2 gRNA and 3’UTR were first labelled with the identical Oligopaints RNA FISH library as in S7 Fig. Subsequently, DENV2 gRNA was detected with Alexa Fluor 647 conjugated secondary oligos, while DENV2 3’UTR was fluorescently detected by labelling with ATTO 565 conjugated secondary oligos. (C) Ratios of DENV2 3’UTR:gRNA fluorescence signals as detected via RNA-FISH in DENV2 NGC infected C6/36 cells and EVs from *Ae*. *aegypti* saliva (left). Ratios of DENV2 3’UTR:gRNA fluorescence signals as detected via RNA-FISH EVs from *Ae*. *aegypti* saliva from the 3 independent labelling experiments (right). ****, p-value ≤ 10^−4^ as determined by a two-sided Mann-Whitney U test. Exact p-value = 3.74 x 10^−8^. N_Cells_ = 30; N_EVs_ = 28.
(TIF)

S9 Fig (Related to Fig 3). **Barcoded DENV2 RNA FISH Oligos specifically label their respective viral RNA targets.** Representative images of nuclei (blue), gRNA (magenta), and DENV2 3’UTR (green) in C6/36 cells 2h post infection (MOI = 0.3) with DENV2 NGC and 2h post transfection with DENV2 PR2B 3’ UTR RNA. Scale bar, 50 μm. DENV2 gRNA and 3’UTR were first hybridized with our Oligopaints RNA FISH library (S7 Fig) with barcodes for the DENV2 gRNA and 3’UTR. Subsequently, DENV2 gRNA was visualized with Alexa Fluor 647 conjugated secondary oligos, while DENV2 3’UTR was visualized with ATTO 565 conjugated secondary oligos.
(TIF)

S10 Fig (Related to Fig 3). **Non-specific RNA-FISH labeling of mosquito salivary EVs in mock (PBS) infected Ae. aegypti saliva samples.** Prior to RNA-FISH, we performed RNA extraction followed by subsequent cDNA synthesis and RT-PCR of all mosquito saliva samples imaged using primers targeting DENV2 NS3 (S2 Table). PCR amplicons were not detected in the mock (PBS) infected *Ae*. *aegypti* saliva sample. (A) Image of an EV in brightfield (grey), DENV2 gRNA (green), and DENV2 3’UTR (magenta) from *Ae*. *aegypti* saliva collected 10 d.p.i. after inoculation with PBS. Scale bar, 2 μm. DENV2 gRNA was labelled with ATTO 565 conjugated secondary oligos, while DENV2 3’UTR was labelled with Alexa Fluor 647 conjugated secondary oligos. (B) Image of an EV in brightfield (grey), DENV2 gRNA (magenta), and DENV2 3’UTR (green) from *Ae*. *aegypti* saliva collected 10 d.p.i. after inoculation with PBS. Scale bar, 2 μm. DENV2 gRNA was labelled with an Alexa Fluor 647 conjugated secondary oligos, while DENV2 3’UTR was labelled with an ATTO 565 conjugated secondary oligos. Since the ATTO 565 fluorophore was observed to potentially produce non-specific signal and thus could account for the high 3’UTR:gRNA labelling ratio in the swapped dye experiments in S8C Fig, we recalculated the ratios excluding two EVs that had ratios higher than 3 (outliers in S8C Fig) and this rigorous analysis still showed a significantly higher 3’UTR:gRNA ratios in EVs relative to cells (median 3’UTR:gRNA ratio of 0.619 (± 0.513) in C6/36 cells, and 1.44 (± 0.584) in saliva EVs; p = 1.55x10^-7^ as determined by a two-sided Mann-Whitney U test).
(TIF)

S11 Fig (Related to Fig 3). **Determining apparent diameter of salivary EVs visualized by RNA-FISH.** (A) To determine the apparent diameter of gRNA and 3’UTR labelled salivary EVs visualized by confocal microscopy, we first used their Brightfield (BF) images. For this analysis diameters were measured by two individuals (DC and TS) independently. To ensure that the same EVs were being analyzed by both individuals, we only analyzed images with 1 EV/image. No significant differences in apparent diameters of EVs used for analysis in Fig 3 (left) using BF images as determined by DC (median diameter = 0.621 μm ± 0.147 μm) or TS (median diameter = 0.614 μm ± 0.107 μm), p = 0.429 as determined by a Student’s t-test with Welch’s correction. No significant differences in apparent diameter of EVs used for analysis in S8 Fig (right) using BF images as determined by DC (median diameter = 0.766 μm ± 0.132 μm) or TS (median diameter = 0.708 μm ± 0.113 μm), p = 0.124 as determined by a Student’s t-test with Welch’s correction. (B) Since there were no significant differences in diameters of EVs determined via DC compared to TS, we proceeded to compare the apparent diameter of EVs as determined by TS via BF compared to diameter determined using 647 fluorescence (Fluor). There were no significant difference in apparent diameters of EVs used for analysis in Fig 3 (left) using BF compared to 647 Fluor images (median diameter BF_TS_ = 0.614 μm ± 0.107 μm, median diameter Fluor_TS_ = 0.647 ± 0.194 μm), p = 0.297 as determined by a Student’s t-test with Welch’s correction. There was a modest but significant increase in apparent diameters of EVs used for analysis in S8 Fig (right) using BF compared to 647 Fluor images (median diameter BF_TS_ = 0.708 μm ± 0.113 μm, median diameter Fluor_TS_ = 0.583 ± 0.123 μm), p = 0.013 as determined by a Student’s t-test with Welch’s correction. (C) To exclude the possibility that we were including diffraction limited objects in our analysis, which could potentially be DENV2 virions or small EVs, we measured the apparent diameter of 100 nm diameter fluorescent Tetraspeck nanospheres as determined by BF and 647 fluorescence images. We proceeded to use these mean values + one standard deviation as a cutoff to exclude EVs at or below these values in our analysis. The apparent diameter of the nanospheres in confocal microscopy was significantly larger using 647 fluorescence (Fluor) compared to BF images (median diameter BF_Tetraspeck nanospheres_ = 0.420 μm ± 0.052 μm; median diameter Fluor_Tetraspeck nanospheres_ = 0.576 μm ± 0.049 μm), p = 2.2 x10^-16^ as determined by a Student’s t-test with Welch’s correction. (D) Importantly, even after applying these stringent cutoff criteria, the median ratios of 3’UTR: gRNA fluorescence in EVs as determined by DC or TS independently using the same EV dataset as in (A) did not change dramatically. N_Tetraspeck nanospheres_ = 33. *, p-value ≤ 0.05; ****, p-value ≤ 10^−4^ as determined by a Student’s t-test with Welch’s correction.
(TIF)

S12 Fig (Related to Fig 4). **The antibody against dsRNA does not recognize sfRNA.** Huh-7 cells were transfected with *in vitro*-transcribed sfRNA and intracellular sfRNA was quantified by RT-qPCR or cells were stained with the anti-dsRNA J2 antibody J2. (A) The table shows the amount of intracellular sfRNA per cell. (B) Detection with the anti-dsRNA antibody in mock-infected cells, cells infected for 2 days with DENV2 NGC at MOI of 1, and cells 24 hours post sfRNA transfection. Representative images are shown. Of note, sfRNA transfection and infection were conducted at the same time; however, the sfRNA transfected cells were analyzed 1 day after transfection, whereas the infected cells were analyzed 2 days later. This time difference may explain the variation in the size of nuclei.
(TIF)

S13 Fig (Related to Fig 4). **Additional repeat for Huh-7 cells supplemented with PR1940- and PR9963-infected saliva.** (A-D) Representative pictures of nuclei (DAPI, blue) and dsRNA (green) from (A, C), number of dsRNA foci per (B, D) Huh-7 cell supplemented with saliva infected with DENV2 PR1940 or PR9963 containing either high or low sfRNA:gRNA ratio. (A, C) Scale bar, 50 μm. (B, D) Each point indicates dsRNA foci in one cell. Bars show geometric means ± 95% C.I..
(TIF)

S14 Fig (Related to Fig 4). **The percentage of dsRNA foci positive Huh-7 cells per image in PR6452 (A), PR1940-1 (B) PR1940-2 (C) and PR9963 (D) infected saliva inoculation.** Each point indicates the percentage of dsRNA positive Huh-7 cell in one image. Bars show mean ± s.e.m.
(TIF)

S15 Fig (Related to Fig 4). **LoD and equation for absolute quantification of DENV2 gRNA with primers targeting NS5.** *In vitro* transcribed RT-qPCR target was serially diluted before quantification by RT-qPCR. (**A**) Fractions of positive replicates were plotted against gRNA copies. At 95% confidence, LoD of log2 DENV2 gRNA is less than 1.5, equaling 2.8 copies. Points show means ± s.e.m. from six technical replicates from two independent experiments. (**B**) Standard curve for absolute quantification of gRNA copies. Dilutions ranged from 6 x 10^8^ to 117 copies. Points show means from six technical replicates from two independent experiments.
(TIF)

S16 Fig (Related to Fig 4). **(A)** gRNA copies in primary dermal fibroblasts incubated with DENV2 NGC-infected saliva inoculum with either low or high sfRNA:gRNA ratio. p-value as determined by unpaired t-test. **(B)** Same data as in (**A**) except gRNA copies is plotted vs sfRNA:gRNA ratio of the DENV2 infected saliva samples.
(TIF)

S17 Fig (Related to Fig 5). Absolute gRNA copy numbers in supernatants of Huh-7 cells pre-treated with sense and antisense DENV2 3’UTR prior to infection. p-values as determined by an unpaired t-test with Welch’s correction. Points demonstrate means from two independent experiments. Bars show mean ± s.e.m.
(TIF)

S18 Fig (Related to Fig 1B). Uncropped northern blot of sfRNA species detected in mock and DENV2 infected Huh-7 cells(left) and mosquito saliva (right). 2 μg of total cellular RNA from mock or MOI = 2 DENV2 infected Huh-7 cells was loaded onto a denaturing acrylamide gel for visualization. Total RNA of 30 pooled mosquito saliva samples from mock (PBS) or DENV2 inoculated mosquitoes was loaded onto a denaturing acrylamide gel for visualization.
(TIF)

S19 Fig (Related to Fig 5). Standard curve used for absolute quantification of gRNA copy numbers. Dilutions of standard RNA ranged from 1.99 x 10^10^ to 199 copies. Points demonstrate means from six technical replicates from two independent experiments.
(TIF)

S1 Table (Related to Fig 4). **Conditions of the saliva inoculum for dermal fibroblasts**.
(DOCX)

S2 Table Oligonucleotides used for RT-qPCR. (DOCX)

## References

[1] Pierson TC, Diamond MS. The continued threat of emerging flaviviruses. Nature Microbiology. 2020;5(6):796–812. Epub 2020/05/06. doi: 10.1038/s41564-020-0714-0 ; PubMed Central PMCID: PMC7696730. 32367055 PMC7696730

[2] Wichit S, Ferraris P, Choumet V, Missé D. The effects of mosquito saliva on dengue virus infectivity in humans. Current Opinion in Virology. 2016;21:139–45. doi: 10.1016/j.coviro.2016.10.001 27770704

[3] Cox J, Mota J, Sukupolvi-Petty S, Diamond MS, Rico-Hesse R. Mosquito bite delivery of dengue virus enhances immunogenicity and pathogenesis in humanized mice. Journal of Virology. 2012;86(14):7637–49. doi: 10.1128/JVI.00534-12 22573866 PMC3416288

[4] McCracken MK, Gromowski GD, Garver LS, Goupil BA, Walker KD, Friberg H, et al. Route of inoculation and mosquito vector exposure modulate dengue virus replication kinetics and immune responses in rhesus macaques. PLoS Neglected Tropical Diseases. 2020;14(4):e0008191. Epub 2020/04/09. doi: 10.1371/journal.pntd.0008191 ; PubMed Central PMCID: PMC7141610 32267846 PMC7141610

[5] Jin L, Guo X, Shen C, Hao X, Sun P, Li P, et al. Salivary factor LTRIN from *Aedes aegypti* facilitates the transmission of Zika virus by interfering with the lymphotoxin-beta receptor. Nature Immunology. 2018;19(4):342–53. Epub 2018/03/07. doi: 10.1038/s41590-018-0063-9 . 29507355

[6] Sun P, Nie K, Zhu Y, Liu Y, Wu P, Liu Z, et al. A mosquito salivary protein promotes flavivirus transmission by activation of autophagy. Nature Communications. 2020;11(1):260. Epub 2020/01/16. doi: 10.1038/s41467-019-14115-z ; PubMed Central PMCID: PMC6959235. 31937766 PMC6959235

[7] Pijlman GP, Funk A, Kondratieva N, Leung J, Torres S, van der Aa L, et al. A highly structured, nuclease-resistant, noncoding RNA produced by flaviviruses is required for pathogenicity. Cell Host & Microbe. 2008;4(6):579–91. Epub 2008/12/10. doi: 10.1016/j.chom.2008.10.007 . 19064258

[8] Bidet K, Dadlani D, Garcia-Blanco MA. G3BP1, G3BP2 and CAPRIN1 are required for translation of interferon stimulated mRNAs and are targeted by a dengue virus non-coding RNA. PLoS Pathogens. 2014;10(7). doi: 10.1371/journal.ppat.1004242 24992036 PMC4081823

[9] Slonchak A, Khromykh AA. Subgenomic flaviviral RNAs: What do we know after the first decade of research. Antiviral Research. 2018;159:13–25. Epub 2018/09/16. doi: 10.1016/j.antiviral.2018.09.006 . 30217649

[10] Slonchak A, Wang X, Aguado J, Sng JDJ, Chaggar H, Freney ME, et al. Zika virus noncoding RNA cooperates with the viral protein NS5 to inhibit STAT1 phosphorylation and facilitate viral pathogenesis. Sci Adv. 2022;8(48):eadd8095. Epub 20221130. doi: 10.1126/sciadv.add8095 ; PubMed Central PMCID: PMC9710884. 36449607 PMC9710884

[11] Manokaran G, Finol E, Wang C, Gunaratne J, Bahl J, Ong EZ, et al. Dengue subgenomic RNA binds TRIM25 to inhibit interferon expression for epidemiological fitness. Science. 2015;350(6257):217–21. Epub 2015/07/04. doi: 10.1126/science.aab3369 ; PubMed Central PMCID: PMC4824004. 26138103 PMC4824004

[12] Michalski D, Ontiveros JG, Russo J, Charley PA, Anderson JR, Heck AM, et al. Zika virus noncoding sfRNAs sequester multiple host-derived RNA-binding proteins and modulate mRNA decay and splicing during infection. Journal of Biological Chemistry. 2019;294(44):16282–96. doi: 10.1074/jbc.RA119.009129 31519749 PMC6827284

[13] Pompon J, Manuel M, Ng GK, Wong B, Shan C, Manokaran G, et al. Dengue subgenomic flaviviral RNA disrupts immunity in mosquito salivary glands to increase virus transmission. PLoS Pathogens. 2017;13(7):e1006535. Epub 2017/07/29. doi: 10.1371/journal.ppat.1006535 ; PubMed Central PMCID: PMC5555716. 28753642 PMC5555716

[14] Slonchak A, Hugo LE, Freney ME, Hall-Mendelin S, Amarilla AA, Torres FJ, et al. Zika virus noncoding RNA suppresses apoptosis and is required for virus transmission by mosquitoes. Nature Communications. 2020;11(1):1–14. doi: 10.1038/s41467-020-16086-y 32371874 PMC7200751

[15] Bennett SN, Holmes EC, Chirivella M, Rodriguez DM, Beltran M, Vorndam V, et al. Molecular evolution of dengue 2 virus in Puerto Rico: positive selection in the viral envelope accompanies clade reintroduction. Journal of General Virology. 2006;87(Pt 4):885–93. Epub 2006/03/11. doi: 10.1099/vir.0.81309-0 . 16528038

[16] Filomatori CV, Carballeda JM, Villordo SM, Aguirre S, Pallares HM, Maestre AM, et al. Dengue virus genomic variation associated with mosquito adaptation defines the pattern of viral non-coding RNAs and fitness in human cells. PLoS Pathogens. 2017;13(3):e1006265. Epub 2017/03/07. doi: 10.1371/journal.ppat.1006265 ; PubMed Central PMCID: PMC5354447. 28264033 PMC5354447

[17] Syenina A, Vijaykrishna D, Gan ES, Tan HC, Choy MM, Siriphanitchakorn T, et al. Positive epistasis between viral polymerase and the 3′ untranslated region of its genome reveals the epidemiologic fitness of dengue virus. Proceedings of the National Academy of Sciences of the United States of America. 2020;117(20):11038–47. doi: 10.1073/pnas.1919287117 32366663 PMC7245076

[18] Yeh SC, Diosa-Toro M, Tan WL, Rachenne F, Hain A, Yeo CPX, et al. Characterization of dengue virus 3’UTR RNA binding proteins in mosquitoes reveals that AeStaufen reduces subgenomic flaviviral RNA in saliva. PLoS Pathog. 2022;18(9):e1010427. Epub 20220919. doi: 10.1371/journal.ppat.1010427 ; PubMed Central PMCID: PMC9531803. 36121894 PMC9531803

[19] Kołat D, Hammouz R, Bednarek AK, Płuciennik E. Exosomes as carriers transporting long non-coding RNAs: Molecular characteristics and their function in cancer. Molecular Medicine Reports. 2019;20(2):851–62. doi: 10.3892/mmr.2019.10340 31173220 PMC6625196

[20] Maori E, Navarro IC, Boncristiani H, Seilly DJ, Rudolph KLM, Sapetschnig A, et al. A Secreted RNA Binding Protein Forms RNA-Stabilizing Granules in the Honeybee Royal Jelly. Molecular Cell. 2019;74(3):598–608 e6. Epub 2019/05/06. doi: 10.1016/j.molcel.2019.03.010 ; PubMed Central PMCID: PMC6509358. 31051140 PMC6509358

[21] Vora A, Zhou W, Londono-Renteria B, Woodson M, Sherman MB, Colpitts TM, et al. Arthropod EVs mediate dengue virus transmission through interaction with a tetraspanin domain containing glycoprotein Tsp29Fb. Proceedings of the National Academy of Sciences of the United States of America. 2018;115(28):E6604–E13. doi: 10.1073/pnas.1720125115 29946031 PMC6048473

[22] Reyes-Ruiz JM, Osuna-Ramos JF, De Jesus-Gonzalez LA, Hurtado-Monzon AM, Farfan-Morales CN, Cervantes-Salazar M, et al. Isolation and characterization of exosomes released from mosquito cells infected with dengue virus. Virus Research. 2019;266:1–14. Epub 2019/04/02. doi: 10.1016/j.virusres.2019.03.015 . 30930201

[23] He F, Liu W, Zheng S, Zhou L, Ye B, Qi Z. Ion transport through dimethyl sulfoxide (DMSO) induced transient water pores in cell membranes. Molecular Membrane Biology. 2012;29(3–4):107–13. doi: 10.3109/09687688.2012.687460 22656651

[24] Lok SM, Costin JM, Hrobowski YM, Hoffmann AR, Rowe DK, Kukkaro P, et al. Release of dengue virus genome induced by a peptide inhibitor. PLoS One. 2012;7(11):e50995. Epub 2012/12/12. doi: 10.1371/journal.pone.0050995 ; PubMed Central PMCID: PMC3511436. 23226444 PMC3511436

[25] Nawaz M, Malik MI, Zhang H, Hassan IA, Cao J, Zhou Y, et al. Proteomic Analysis of Exosome-Like Vesicles Isolated From Saliva of the Tick Haemaphysalis longicornis. Front Cell Infect Microbiol. 2020;10:542319. Epub 20201022. doi: 10.3389/fcimb.2020.542319 ; PubMed Central PMCID: PMC7642894. 33194791 PMC7642894

[26] Beliveau BJ, Joyce EF, Apostolopoulos N, Yilmaz F, Fonseka CY, McCole RB, et al. Versatile design and synthesis platform for visualizing genomes with Oligopaint FISH probes. Proc Natl Acad Sci U S A. 2012;109(52):21301–6. Epub 2012/12/14. doi: 10.1073/pnas.1213818110 ; PubMed Central PMCID: PMC3535588. 23236188 PMC3535588

[27] Beliveau BJ, Kishi JY, Nir G, Sasaki HM, Saka SK, Nguyen SC, et al. OligoMiner provides a rapid, flexible environment for the design of genome-scale oligonucleotide in situ hybridization probes. Proc Natl Acad Sci U S A. 2018;115(10):E2183–E92. Epub 2018/02/22. doi: 10.1073/pnas.1714530115 ; PubMed Central PMCID: PMC5877937. 29463736 PMC5877937

[28] Thery C, Witwer KW, Aikawa E, Alcaraz MJ, Anderson JD, Andriantsitohaina R, et al. Minimal information for studies of extracellular vesicles 2018 (MISEV2018): a position statement of the International Society for Extracellular Vesicles and update of the MISEV2014 guidelines. J Extracell Vesicles. 2018;7(1):1535750. Epub 2019/01/15. doi: 10.1080/20013078.2018.1535750 ; PubMed Central PMCID: PMC6322352. 30637094 PMC6322352

[29] Nolte E, Cremer T, Gallo RC, Margolis LB. Extracellular vesicles and viruses: Are they close relatives? Proceedings of the National Academy of Sciences of the United States of America. 2016;113(33):9155–61. doi: 10.1073/pnas.1605146113 27432966 PMC4995926

[30] Kerviel A, Zhang M, Altan-Bonnet N. A New Infectious Unit: Extracellular Vesicles Carrying Virus Populations. Annu Rev Cell Dev Biol. 2021;37:171–97. Epub 2021/07/17. doi: 10.1146/annurev-cellbio-040621-032416 . 34270326

[31] Diosa-Toro M, Strilets T, Yeh S-C, Garcia-Blanco MA. Tinkering with extracellular vesicles viruses evolve new infectious units. ExRNA. 2022;4.

[32] Zhou W, Woodson M, Sherman MB, Neelakanta G, Sultana H. Exosomes mediate Zika virus transmission through SMPD3 neutral Sphingomyelinase in cortical neurons. Emerg Microbes Infect. 2019;8(1):307–26. Epub 2019/03/15. doi: 10.1080/22221751.2019.1578188 ; PubMed Central PMCID: PMC6455149. 30866785 PMC6455149

[33] Zhou W, Woodson M, Neupane B, Bai F, Sherman MB, Choi KH, et al. Exosomes serve as novel modes of tick-borne flavivirus transmission from arthropod to human cells and facilitates dissemination of viral RNA and proteins to the vertebrate neuronal cells. PLoS Pathogens. 2018;14(1):e1006764. Epub 2018/01/05. doi: 10.1371/journal.ppat.1006764 ; PubMed Central PMCID: PMC5754134. 29300779 PMC5754134

[34] Bukong TN, Momen-Heravi F, Kodys K, Bala S, Szabo G. Exosomes from hepatitis C infected patients transmit HCV infection and contain replication competent viral RNA in complex with Ago2-miR122-HSP90. PLoS Pathog. 2014;10(10):e1004424. Epub 2014/10/03. doi: 10.1371/journal.ppat.1004424 ; PubMed Central PMCID: PMC4183590. 25275643 PMC4183590

[35] York SB, Sun L, Cone AS, Duke LC, Cheerathodi MR, Meckes DG Jr,. Zika Virus Hijacks Extracellular Vesicle Tetraspanin Pathways for Cell-to-Cell Transmission. mSphere. 2021:e0019221. Epub 2021/07/01. doi: 10.1128/mSphere.00192-21 ; PubMed Central PMCID: PMC8265634. 34190582 PMC8265634

[36] Bidet K, Garcia-Blanco MA. Flaviviral RNAs: weapons and targets in the war between virus and host. Biochemical Journal. 2014;462(2):215–30. doi: 10.1042/BJ20140456 25102029

[37] Buck AH, Coakley G, Simbari F, McSorley HJ, Quintana JF, Le Bihan T, et al. Exosomes secreted by nematode parasites transfer small RNAs to mammalian cells and modulate innate immunity. Nature Communications 2014;5:5488. Epub 2014/11/26. doi: 10.1038/ncomms6488 ; PubMed Central PMCID: PMC4263141. 25421927 PMC4263141

[38] Cai Q, Qiao L, Wang M, He B, Lin F-M, Palmquist J, et al. Plants send small RNAs in extracellular vesicles to fungal pathogen to silence virulence genes. Science. 2018;360(6393):1126–9. doi: 10.1126/science.aar4142 29773668 PMC6442475

[39] Rosen L, Gubler D. The use of mosquitoes to detect and propagate dengue viruses. Am J Trop Med Hyg. 1974;23(6):1153–60. Epub 1974/11/01. doi: 10.4269/ajtmh.1974.23.1153 . 4429185

[40] Bischoff V, Vignal C, Boneca IG, Michel T, Hoffmann JA, Royet J. Function of the drosophila pattern-recognition receptor PGRP-SD in the detection of Gram-positive bacteria. Nature Immunology. 2004;5(11):1175. doi: 10.1038/ni1123 15448690

[41] Forootan A, Sjöback R, Björkman J, Sjögreen B, Linz L, Kubista M. Methods to determine limit of detection and limit of quantification in quantitative real-time PCR (qPCR). Biomolecular Detection and Quantification. 2017;12:1–6. doi: 10.1016/j.bdq.2017.04.001 28702366 PMC5496743

[42] Thery C, Amigorena S, Raposo G, Clayton A. Isolation and characterization of exosomes from cell culture supernatants and biological fluids. Curr Protoc Cell Biol. 2006;Chapter 3:Unit 3 22. doi: 10.1002/0471143030.cb0322s30 . 18228490

[43] Langmead B, Salzberg SL. Fast gapped-read alignment with Bowtie 2. Nat Methods. 2012;9(4):357–9. Epub 2012/03/06. doi: 10.1038/nmeth.1923 ; PubMed Central PMCID: PMC3322381. 22388286 PMC3322381

[44] Marcais G, Kingsford C. A fast, lock-free approach for efficient parallel counting of occurrences of k-mers. Bioinformatics. 2011;27(6):764–70. Epub 2011/01/11. doi: 10.1093/bioinformatics/btr011 ; PubMed Central PMCID: PMC3051319. 21217122 PMC3051319

[45] Nir G, Farabella I, Perez Estrada C, Ebeling CG, Beliveau BJ, Sasaki HM, et al. Walking along chromosomes with super-resolution imaging, contact maps, and integrative modeling. PLoS Genet. 2018;14(12):e1007872. Epub 2018/12/27. doi: 10.1371/journal.pgen.1007872 ; PubMed Central PMCID: PMC6324821. 30586358 PMC6324821

[46] Nguyen HQ, Chattoraj S, Castillo D, Nguyen SC, Nir G, Lioutas A, et al. 3D mapping and accelerated super-resolution imaging of the human genome using in situ sequencing. Nat Methods. 2020;17(8):822–32. Epub 2020/07/29. doi: 10.1038/s41592-020-0890-0 ; PubMed Central PMCID: PMC7537785. 32719531 PMC7537785

[47] Schneider CA, Rasband WS, Eliceiri KW. NIH Image to ImageJ: 25 years of image analysis. Nat Methods. 2012;9(7):671–5. Epub 2012/08/30. doi: 10.1038/nmeth.2089 ; PubMed Central PMCID: PMC5554542. 22930834 PMC5554542

[48] Team R. RStudio: Integrated Development Environment for R. Boston, MA2021.

