# Supplementary material for: The anti-immune dengue subgenomic flaviviral RNA is present in vesicles in mosquito saliva and is associated with increased infectivity
Source: PLoS Pathog. 2023 Mar 30;19(3):e1011224. doi: 10.1371/journal.ppat.1011224 (PMC10062553; doi:10.1371/journal.ppat.1011224)
Supplement: S1 Table — Conditions of the saliva inoculum for dermal fibroblasts. (DOCX) [file ppat.1011224.s020.docx]

S1 Table. Conditions of the saliva inocula for dermal fibroblasts.

| Repeat (High or low sfRNA ratio) | Date of saliva collection | gRNA (copies/µl) | sfRNA (copies/µl) | sfRNA:gRNA ratio | Total volume of saliva added per well (µl) | gRNA inoculum (copies/well) | gRNA copies per well |
| --- | --- | --- | --- | --- | --- | --- | --- |
|  |  |  |  |  |  |  |  |
| Low-1 | 20/10/2021 | 1.6 x10^2^ | 1.1 x10^3^ | 6.9 | 25.00  (23.88 inf. + 1.12 uninf. ) | 3.9 x10^3^ | Not detected |
| Low-2 | 18/10/2021 | 2.0 x10^2^ | 2.0 x10^3^ | 10.1 | 25.00  (19.6 inf. + 5.4 uninf. ) | 3.9 x10^3^ | Not detected |
| Low-3 | 20/10/2021 | 2.5 x10^2^ | 2.6 x10^3^ | 10.1 | 25.00  (15.3 inf. + 9.7 uninf. ) | 3.9 x10^3^ | Not detected |
| High-1 | 20/10/2021 | 3.5 x10^2^ | 4.7 x10^3^ | 13.5 | 25.00  (11.05 inf. + 13.95 uninf. ) | 3.9 x10^3^ | 101 |
| High-2 | 18/10/2021 | 4.0 x10^2^ | 6.2 x10^3^ | 15.7 | 25.00  (9.80 inf. + 15.20 uninf.) | 3.9 x10^3^ | 141 |
| High-3 | 18/102021 | 1.6 x10^2^ | 3.3 x10^3^ | 21 | 25.00  (25.00 inf. + 0 uninf. ) | 3.9 x10^3^ | 319 |
