# Supplementary material for: The anti-immune dengue subgenomic flaviviral RNA is present in vesicles in mosquito saliva and is associated with increased infectivity
Source: PLoS Pathog. 2023 Mar 30;19(3):e1011224. doi: 10.1371/journal.ppat.1011224 (PMC10062553; doi:10.1371/journal.ppat.1011224)
Supplement: S2 Table — (DOCX) [file ppat.1011224.s021.docx]

Table S2. Primer sets used for Real-Time qPCR in figures

| Gene name | Forward primer | Reverse primer | Probe | Target | Figure usage | Reference |
| --- | --- | --- | --- | --- | --- | --- |
| E | CAGGTTATGGCACTGTCACGAT | CCATCTGCAGCAACACCATCTC | CTCTCCGAGAACAGGCCTCGACTTAAA | Envelope | Figure 1, 2 and Figure 4 | [1] |
| NS5 | CTCCCTGAGTGGAGTGGAAG | ACACGCACCACCTTGTTTTG |  | NS5 | S15 and S16 Figs |  |
| NS5 | CCATGAAAAGATTCAGAAG | GCTGCGATTTGTAAGGG |  | NS5 | Figure 5 | [2] |
| 3’UTR/sfRNA1 | GTGAGCCCCGTCCAAGG | GCTGCGATTTGTAAGGG |  | 3’UTR or sfRNA | Figure 1, 2, 4 and Figure 5 | [2] |
| DENV NS3 | GAA ATG GGT GCC AAC TTC AAG GCT | TCT TTG TGC TGC ACT AGA GTG GGT |  | NS3 | S10 Fig | [3] |
| GAPDH | AGC CAC ATC GCT CAG ACA C | GCC CAA TAC GAC CAA ATC C |  | Human GAPDH | Figure 5 |  |
| IFN-β | GAG CTA CAA CTT GCT TGG ATT C | CAA GCC TCC CAT TCA ATT GC |  | Human IFN-β | Figure 5 | [4] |
| IFN-λ_1_ | CGC CTT GGA AGA GTC ACT CA | GAA GCC TCA GGT CCC AAT TC |  | Human  IFN-λ_1_ | Figure 5 | [4] |
| ISG15 | GAG AGG CAG CGA ACT CAT CT | CTT CAG CTC TGA CAC CGA CA |  | Human  ISG15 | Figure 5 | [5] |
| MX-1 | TTC AGC ACC TGA TGG CCT ATC | TGG ATG ATC AAA GGG ATG TGG |  | Human  MX-1 | Figure 5 | [6] |

1. Johnson BW, Russell BJ, Lanciotti RS. Serotype-specific detection of dengue viruses in a fourplex real-time reverse transcriptase PCR assay. J Clin Microbiol. 2005;43(10):4977-83. Epub 2005/10/07. doi: 10.1128/JCM.43.10.4977-4983.2005. PubMed PMID: 16207951; PubMed Central PMCID: PMCPMC1248506.

2. Bidet K, Dadlani D, Garcia-Blanco MA. G3BP1, G3BP2 and CAPRIN1 are required for translation of interferon stimulated mRNAs and are targeted by a dengue virus non-coding RNA. PLoS Pathogens. 2014;10(7). doi: 10.1371/journal.ppat.1004242.

3. Phillips SL, Soderblom EJ, Bradrick SS, Garcia-Blanco MA. Identification of Proteins Bound to Dengue Viral RNA In Vivo Reveals New Host Proteins Important for Virus Replication. mBio. 2016;7(1):e01865-15. Epub 2016/01/07. doi: 10.1128/mBio.01865-15. PubMed PMID: 26733069; PubMed Central PMCID: PMCPMC4725007.

4. Bayer A, Lennemann NJ, Ouyang Y, Bramley JC, Morosky S, Marques ET, Jr., et al. Type III Interferons Produced by Human Placental Trophoblasts Confer Protection against Zika Virus Infection. Cell Host Microbe. 2016;19(5):705-12. Epub 20160405. doi: 10.1016/j.chom.2016.03.008. PubMed PMID: 27066743; PubMed Central PMCID: PMCPMC4866896.

5. Bektas N, Noetzel E, Veeck J, Press MF, Kristiansen G, Naami A, et al. The ubiquitin-like molecule interferon-stimulated gene 15 (ISG15) is a potential prognostic marker in human breast cancer. Breast Cancer Res. 2008;10(4):R58. Epub 20080715. doi: 10.1186/bcr2117. PubMed PMID: 18627608; PubMed Central PMCID: PMCPMC2575531.

6. Holzinger D, Jorns C, Stertz S, Boisson-Dupuis S, Thimme R, Weidmann M, et al. Induction of MxA gene expression by influenza A virus requires type I or type III interferon signaling. J Virol. 2007;81(14):7776-85. Epub 20070509. doi: 10.1128/JVI.00546-06. PubMed PMID: 17494065; PubMed Central PMCID: PMCPMC1933351.
